# Balancing intestinal and systemic inflammation through cell type-specific expression of the aryl hydrocarbon receptor repressor

**DOI:** 10.1038/srep26091

**Published:** 2016-05-17

**Authors:** Olga Brandstätter, Oliver Schanz, Julia Vorac, Jessica König, Tetsushi Mori, Toru Maruyama, Markus Korkowski, Thomas Haarmann-Stemmann, Dorthe von Smolinski, Joachim L. Schultze, Josef Abel, Charlotte Esser, Haruko Takeyama, Heike Weighardt, Irmgard Förster

**Affiliations:** 1 Immunology and Environment, Life and Medical Sciences (LIMES) Institute, University of Bonn, Carl-Troll-Straße 31, 53115 Bonn, Germany; 2 IUF-Leibniz Research Institute for Environmental Medicine gGmbH, Auf´m Hennekamp 50, 40225 Düsseldorf, Germany; 3 Center for Advanced Biomedical Sciences (TWIns), Waseda University, 2-2, Wakamatsu-cho, Shinjuku-ku, 162-8480, Tokyo, Japan; 4 Institut für Tierpathologie der FU Berlin, Robert von Ostertag Strasse 15, 14163 Berlin; 5 Genomics and Immunoregulation, Life and Medical Sciences (LIMES) Institute, University of Bonn, Carl-Troll-Straße 31, 53115 Bonn, Germany

## Abstract

As a sensor of polyaromatic chemicals the aryl hydrocarbon receptor (AhR) exerts an important role in immune regulation besides its requirement for xenobiotic metabolism. Transcriptional activation of AhR target genes is counterregulated by the AhR repressor (AhRR) but the exact function of the AhRR *in vivo* is currently unknown. We here show that the AhRR is predominantly expressed in immune cells of the skin and intestine, different from other AhR target genes. Whereas AhRR antagonizes the anti-inflammatory function of the AhR in the context of systemic endotoxin shock, AhR and AhRR act in concert to dampen intestinal inflammation. Specifically, AhRR contributes to the maintenance of colonic intraepithelial lymphocytes and prevents excessive IL-1β production and Th17/Tc17 differentiation. In contrast, the AhRR enhances IFN-γ-production by effector T cells in the inflamed gut. Our findings highlight the physiologic importance of cell-type specific balancing of AhR/AhRR expression in response to microbial, nutritional and other environmental stimuli.

The aryl hydrocarbon receptor (AhR) is well known as a ligand-activated transcription factor important for xenobiotic metabolism in the liver and other organs. However, AhR not only acts as a sensor for environmental toxins but also for physiological low molecular weight ligands, such as tryptophan derived photoproducts or dietary components^1^,^2^,^3^. In addition to its important role in xenobiotic metabolism, the AhR signaling pathway also exerts essential regulatory functions in immunity^4^,^5^. AhR activation can directly influence the Th17/Treg balance, facilitating either the generation of Treg or that of Th17 cells depending on the disease model, tissue context and type of AhR ligand^6^,^7^,^8^,^9^,^10^,^11^,^12^,^13^. Direct ligand-dependent activation of the AhR *in vitro* was shown to enhance Th17 differentiation^6^,^11^,^14^,^15^,^16^,^17^, whereas AhR activation *in vivo* often has an anti-inflammatory effect^18^,^19^,^20^,^21^. In line with this anti-inflammatory function, AhR-deficient mice are hypersensitive to LPS-induced shock^22^,^23^, inflammatory bowel disease^8^,^24^,^25^ and *Citrobacter rodentium* infection^8^,^26^,^27^. Furthermore, AhR activation was shown to protect from DSS-induced colitis^9^,^19^,^20^,^28^.

To maintain appropriate barrier immunity, the AhR is critically involved in the development and function of innate lymphoid cells (ILC)-3 in the intestine, in particular IL-22-producing NKp46^+^RORγt^+^ ILC3^8^,^26^,^27^. The AhR is essential for c-kit-dependent intraepithelial γδ T cell expansion in small intestine and colon^24^, as well as skin^29^. Furthermore, activation of the AhR was shown to influence the differentiation and activation of DC *in vitro* and *in vivo*^30^,^31^,^32^.

To induce gene expression of classical AhR target genes, the ligand bound AhR translocates into the nucleus, interacts with AhR-nuclear translocator (ARNT) and binds to xenobiotic response elements (XRE) in the promoter regions of target genes, thereby regulating their expression. The AhR is also able to interact directly with the NF-κB family members RelA and RelB^33^ and with steroid receptors^34^, indicating that the AhR interferes with several signalling cascades.

In light of the major impact of AhR stimulation on the regulation of immune responses, we studied the functional importance of feedback inhibition of the AhR by the AhR-repressor (AhRR) which is encoded by a known target gene of the AhR^35^. Like the AhR, the AhRR is a member of the basic helix loop helix Per-ARNT-Sim (PAS) family^36^, but lacks a transactivation domain. Once induced, the AhRR competes with the AhR for dimerization with ARNT. AhRR/ARNT dimers bind XRE but do not initiate transcription and thus efficiently repress AhR-target gene expression^36^,^37^,^38^. It is still unclear, however, whether the regulatory function of AhRR merely works by feedback inhibition of AhR/ARNT activity, or also through interaction with other proteins.

The promoter of the AhRR itself contains several XRE, GC boxes and a NF-κB site, indicating that signals from other transcriptional pathways, potentially involving Sp1, c-Jun or NF-κB, may lead to expression of the AhRR^37^,^39^. Recently generated AhRR-deficient mice showed an upregulation of the AhR-response gene *cyp1A1* in skin, stomach and spleen, while there was no altered *cyp1A1* expression in liver and heart^40^. However, the function of the AhRR in the regulation of immune responses has not been addressed so far.

In order to obtain more insight into the expression and functional role of the AhRR *in vivo,* we generated AhRR-reporter and -knockout mice, which express enhanced green fluorescent protein (EGFP) under control of the endogenous *ahrr* locus. These mice allow efficient identification of AhRR expression at the single cell level. Here, we show that AhRR expression does not strictly mirror AhR expression and activation but is rather regulated in an organ- and cell-type specific manner. Our findings demonstrate that an optimal balance of AhR and AhRR expression maintains immune homeostasis in the intestine and adjusts the strength of the inflammatory response to microbial challenges.

## Results

### Expression of the AhRR in immune cells of barrier organs

For the generation of AhRR-reporter and -knockout mice an EGFP-cassette was inserted into the second exon of the *ahrr* gene, and the third exon was deleted (Supplementary Fig. 1a). Recombinant AhRR/EGFP ES cell clones were analyzed by Southern blot for the presence of the mutant allele (Supplementary Fig. 1b), and germline transmission was proven by PCR (Supplementary Fig. 1c). Successful mutation of the *ahrr* gene was then confirmed by RT-PCR. The WT *ahrr* allele was detected in mesenteric lymph nodes (MLN) and Peyer’s patches (PP) of naive WT and AhRR^E/+^ mice but not in AhRR^E/E^ mice, whereas EGFP message was present in AhRR^E/+^ and AhRR^E/E^ samples only (Supplementary Fig. 1d). AhRR^E/E^ mice are fertile and do not exhibit any obvious anatomic or behavioral abnormalities.

**Figure 1:**
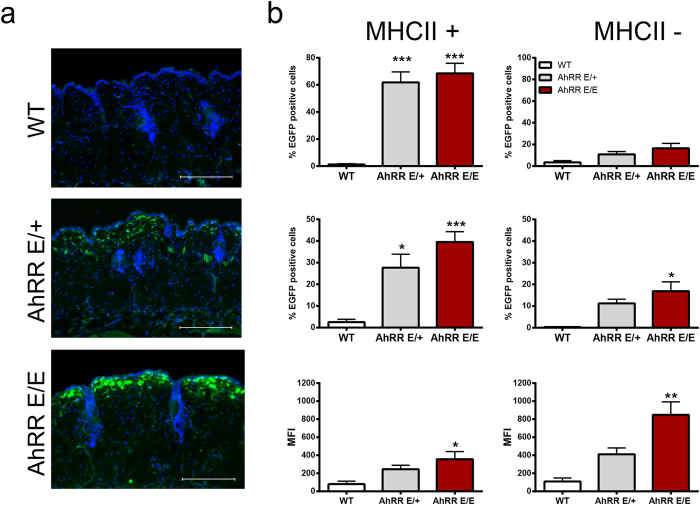
AhRR/EGFP expression in the skin. **(a**) Immunofluorescence analysis of frozen sections of dorsal skin of WT, AhRR^E/+^ and AhRR^E/E^ mice counterstained with DAPI (bars: 200 μm). (**b**) Flow cytometry of single cell suspensions from epidermis (upper panel) and dermis (middle and lower panel) of either MHCII^+^ (left) or MHCII^−^ cells (right) (n = 4). Shown are the percentage of EGFP^+^ cells and the mean fluorescence index (MFI) as indicated. Data are shown as mean ± s.e.m. and significance was determined by one way ANOVA corrected for multiple comparisons by the Sidak method *p < 0.05, **p < 0.01, ***p < 0.001 (WT vs AhRR^E/+^ or AhRR^E/E^ mice).

Expression of the AhRR/EGFP reporter was further analyzed in skin, gut, liver, lung, spleen and lymph nodes (LN) of AhRR^E/+^ and AhRR^E/E^ mice. AhRR was not expressed in liver and only marginally in lung (Supplementary Fig. 1e and data not shown). In skin, expression of AhRR/EGFP was found in the dermis and epidermis of naïve AhRR^E/+^ and AhRR^E/E^ mice (Fig. 1). Expression of AhRR/EGFP could be detected in 60–70% of MHCII^+^ epidermal Langerhans cells (LC) in line with a previous report^41^. In the dermis, 20–40% of MHCII^+^ cells were EGFP^+^ (Fig. 1b). The proportion of AhRR/EGFP-expressing epidermal MHCII^−^ cells, which represent epidermal keratinocytes and T cells, as well as dermal MHCII^−^ cells (fibroblasts and T cells) was 10–20% (Fig. 1b).

**Figure 2:**
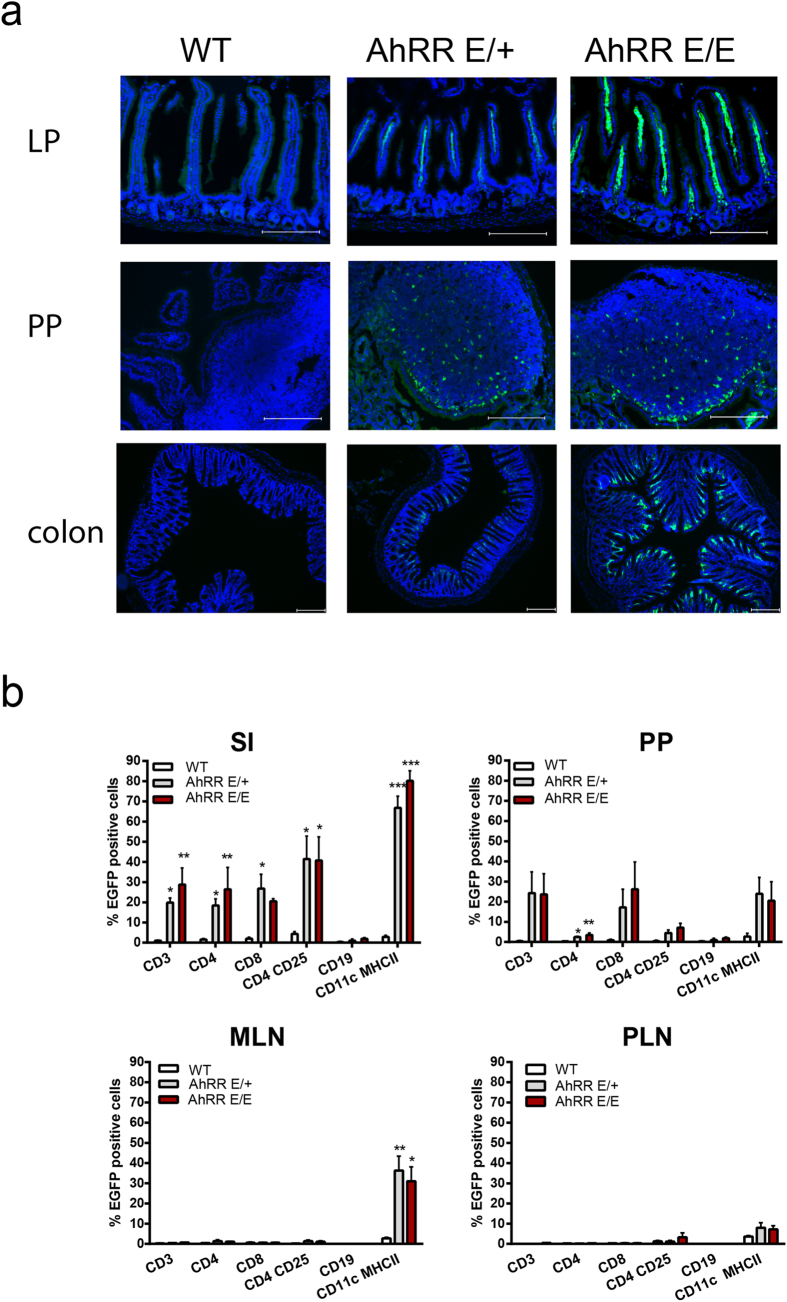
AhRR/EGFP expression in the small intestine and in lymph nodes. **(a)** Immunofluorescence analysis of frozen sections of the SI, PP and colon of WT, AhRR^E/+^ and AhRR^E/E^ mice counterstained with DAPI (bars: 200 μm). (**b**) Flow cytometry of single cell suspensions of the SI LP, PP, MLN and PLN (n = 3–5) of WT, AhRR^E/+^ and AhRR^E/E^ mice. Data are shown as mean ± s.e.m. and significance was determined by one way ANOVA corrected for multiple comparisons by the Sidak method. *p < 0.05, **p < 0.01, ***p < 0.001. (WT vs AhRR^E/+^ or AhRR^E/E^ mice).

Next, we focused on the analysis of AhRR expression in the gut. A large proportion of AhRR/EGFP-expressing cells could be detected in the lamina propria (LP) of the small intestine (SI) in AhRR^E/+^ and AhRR^E/E^ mice (Fig. 2a, top). Furthermore, expression of the AhRR was found in PP, particularly in the dome region (Fig. 2a, middle), and in the LP of the colon (Fig. 2a, bottom). In SI and colon, AhRR expression was not detected in CD45^−^EpCAM^+^ intestinal epithelial cells in contrast to CD45^+^ cells (Supplementary Fig. 2). The intensity of EGFP expression in skin and gut was generally higher in AhRR^E/E^ mice compared to AhRR^E/+^ mice (Figs 1 and 2). This may result from a gene-dosage effect or, alternatively, from the absence of AhRR-mediated suppression of AhR signaling in AhRR^E/E^ animals.

To further quantify expression of the AhRR in different immune cells, T cell subsets, B cells and myeloid cells from the small intestinal LP as well as PP, MLN and peripheral LN (PLN) were analyzed by flow cytometry (Fig. 2b). The highest proportion of AhRR-expressing cells was found in the small intestinal LP. Here, expression of AhRR/EGFP could be detected in 20–40% of all T cell subsets analyzed and in 60–80% of CD11c^+^ MHCII^+^ myeloid cells but not in CD19^+^ B cells. In PP, expression of the AhRR occurs in about 20% of CD8^+^ T cells and CD11c^+^MHCII^+^ cells. In neither MLN nor PLN of naïve mice expression of the AhRR could be detected in T and B cells but in a substantial proportion of CD11c^+^MHCII^+^ cells (Fig. 2b). Specifically, AhRR/EGFP expression was detected in all intestinal DC subsets and was most prominent in CD11b^+^CD103^+^ and CD11b^+^CD103^−^ DC of the SI, CD11b^+^CD103^+^ DC of the colon, and CD11b^−^CD103^+^ as well as CD11b^+^CD103^+^ subsets of the PP and mLN. According to Cerovic *et al*.^42^ intestinal macrophages were identified as F4/80^+^CD64^+^ cells, of which 50–60% were AhRR/EGFP^+^ in both the SI and colon (Supplementary Fig. 3). In spleen, however, no expression of the AhRR was apparent in naïve mice (data not shown).

### Induction of the AhRR by environmental stimuli is mainly AhR-dependent

**Figure 3:**
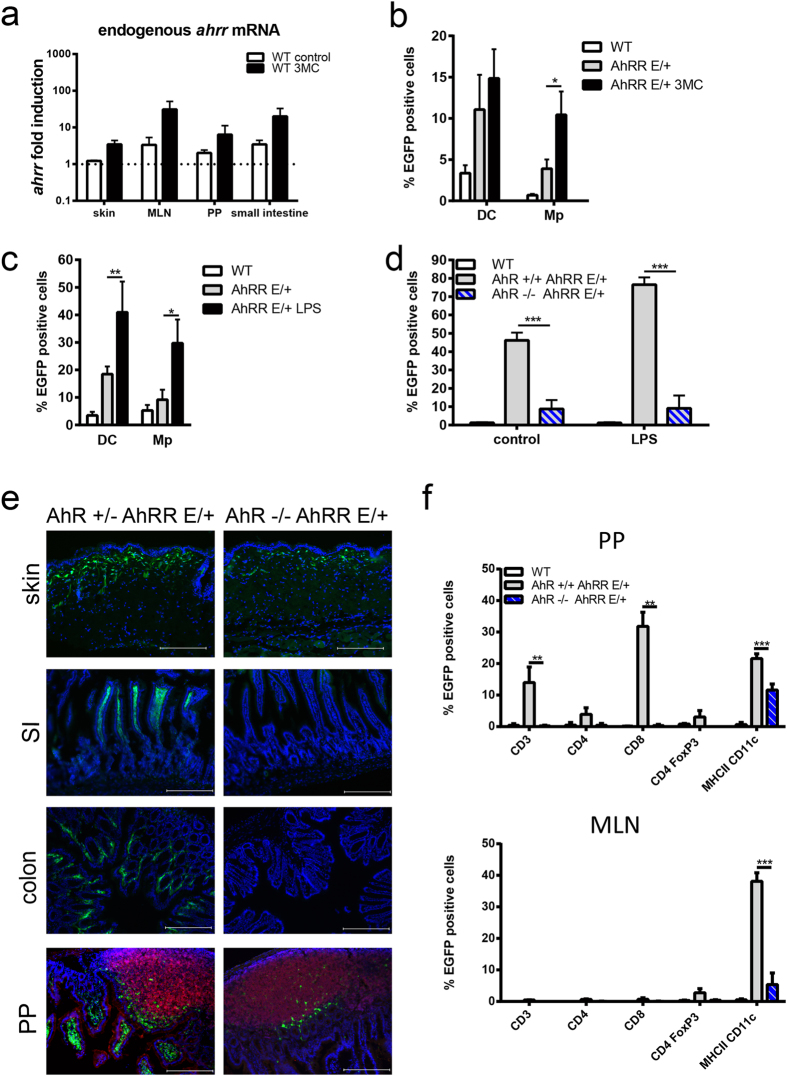
Dependency of *ahrr* expression on AhR signaling. (**a**) *ahRR* mRNA levels in skin, MLN, PP and SI of WT mice (n = 4 mice) that were injected with 3MC in DMSO/olive oil (1:4 v/v) or with solvent alone. **(b**) Percentages of EGFP-expressing cells in peripheral LN of WT and AhRR^E/+^ mice 16 h after i.p. injection of 3MC or solvent (n = 3–8). (**c**) Percentages of EGFP-expressing cells in peripheral LN of WT and AhRR^E/+^ mice 16 h after PBS or LPS injection i.p. (n = 3–4). Data are shown as mean ± s.e.m.; significance was determined by one way ANOVA corrected for multiple comparisons by the Sidak method *p < 0.05 (AhRR^E/+^/LPS vs WT), **p < 0.01 (AhRR^E/+^ vs WT). (**d**) Percentages of EGFP-expressing cells among MHCII^+^CD11c^+^ BMDC of WT, AhR^+/+^ AhRR^E/+^ and AhR^−/−^ AhRR^E/+^ mice stimulated with or w/o LPS (n = 5). Data are shown as mean ± s.e.m.; significance was determined by one way ANOVA corrected for multiple comparisons by the Sidak method, ***p < 0.001 (BMDC from AhR^+/+^AhRR^E/+^ vs AhR^−/−^AhRR^E/+^ mice). **(e**) Immunofluorescence analysis of skin, SI, colon, and PP of AhR^+/−^AhRR^E/+^ and AhR^−/−^AhRR^E/+^ mice (bar: 200 μm) counterstained with DAPI. PP sections were additionally counterstained for B220 expression (red). Data are representative for three independent experiments (**f**) Percentages of EGFP-expressing cells in PP (top) and MLN (bottom) of WT, AhR^+/+^AhRR^E/+^ and AhR^−/−^AhRR^E/+^ mice in different immune cell subsets as indicated. Data are shown as mean ± s.e.m.; significance was determined by one way ANOVA corrected for multiple comparisons by the Sidak method, ***p < 0.001 (AhR^+/+^AhRR^E/+^ vs AhR^−/−^AhRR^E/+^ mice).

To analyze the influence of AhR signaling on the transcription of *ahrr in vivo*, we first measured expression of the endogenous *ahrr* gene by qPCR analyses in WT mice after i.p. application of the high affinity ligand 3-methylcholanthrene (3MC) for 16h. Upregulation of *ahrr* expression appeared higher in MLN and SI, compared with skin and PP after 3MC treatment but the differences were not statistically significant (Fig. 3a). Using the EGFP reporter we also analyzed AhRR expression in T cell subsets and myeloid cells of the SI, PP and MLN after oral 3MC treatment. AhRR expression was significantly upregulated in most of the cell types analyzed in SI and PP, but only in CD4^+^CD25^+^ T cells and CD11c^+^MHCII^+^ myeloid cells of the MLN (Supplementary Fig. 4). Application of 3MC also increased the proportion of AhRR/EGFP^+^ cells in peripheral LN CD11c^+^ DC and F4/80^+^ macrophages notably (Fig. 3b). An even stronger increase of AhRR/EGFP-expressing cells was apparent after treatment of the mice with LPS, a known inducer of AhR expression^22^,^23^ (Fig. 3c).

To investigate the contribution of AhR-signaling to AhRR expression more directly we generated AhR^−/−^AhRR^E/+^ double mutant mice. Already in the absence of intentional stimulation BMDC of AhR^−/−^AhRR^E/+^ mice displayed a strongly reduced frequency of AhRR/EGFP-expressing cells as compared to AhR^+/+^AhRR^E/+^ BMDC, indicating that the AhR indeed controls AhRR expression (Fig. 3d) in line with previous data^43^. The LPS-dependent induction of AhRR expression, however, was no longer visible in AhR-deficient BMDC and thus appeared to be a consequence of LPS-induced AhR activation. Of note, some residual AhRR/EGFP expression was still detectable in AhR^−/−^AhRR^E/+^ BMDC independent of LPS stimulation, indicating that AhRR expression can also be triggered at low levels by an AhR-independent mechanism.

*In vivo,* the representation of EGFP-expressing cells in skin, SI, colon, and PP of AhR^−/−^AHRR^E/+^ mice was markedly reduced compared to AhR^+/−^AhRR^E/+^ mice, indicating that AhR activation in barrier organs strongly contributes to AhRR expression *in vivo* (Fig. 3e). As this AhR-dependent AhRR expression occurs in naïve mice without deliberate application of exogenous AhR ligands, our findings support the assumption that endogenous ligands, such as the tryptophan photoproduct FICZ in the skin, nutritional or microbial AhR-ligands in the gut^3^ might continuously activate the AhR and drive AhRR expression. Nevertheless, a notable residual AhRR/EGFP expression in the absence of the AhR was detected in the skin and in the dome area of PP (Fig. 3e,f). In PP, AhR-independent expression of the AhRR was restricted to MHCII^+^CD11c^+^ cells, and some of these cells were also detected in MLN probably representing migratory DC from the PP (Fig. 3f). Therefore, our data support the conclusion that AhRR expression *in vivo* is mostly but not entirely dependent on environmentally induced AhR signaling.

### Influence of AhRR deficiency on *cyp1a1* expression

To assess the functional effect of AhRR-deficiency, we first tested whether absence of the AhRR influences the expression of a typical AhR-target gene*, cytochrome p450 (cyp) 1A1 in vivo*. Application of 3MC enhanced *cyp1A1* expression in both WT and AhRR^E/E^ mice but this induction was not significantly stronger in AhRR^E/E^ mice compared with WT mice (Supplementary Fig. 5a). In addition, we analyzed *cyp1A1* expression in BMDC after stimulation with 3MC or LPS *in vitro*. Whereas 3MC did not cause a notable upregulation of *cyp1A1* in WT BMDC compared to unstimulated controls, *cyp1A1* was 5fold enhanced in AhRR^E/+^ and about 15fold in AhRR^E/E^ BMDC (Supplementary Fig. 5b). Stimulation with LPS, however, caused only minor changes in *cyp1A1* expression in BMDC of all genotypes (Supplementary Fig. 5b). Taken together, these findings indicate that *cyp1a1* expression is only moderately suppressed by the AhRR depending on cell-type and location.

### AhRR-deficient mice are protected from LPS shock

**Figure 4:**
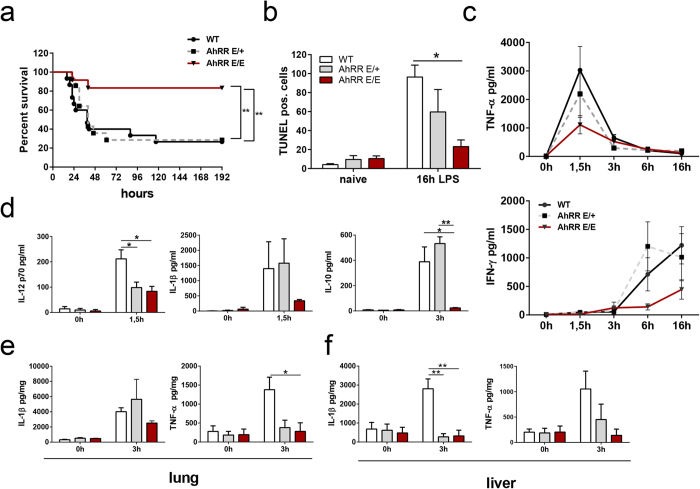
AhRR-deficiency confers enhanced resistance to LPS induced shock. **(a)** Survival curves of AhRR^+/+^, AhRR^E/+^ and AhRR^E/E^ mice injected with 15 mg/kg LPS (n = 12–15) log-rank test **p < 0.01, AhR^+/+^ vs AhRR^E/+^ or AhR^+/+^ vs AhRR^E/E^ mice. (**b**) Numbers of apoptotic cells were determined by TUNEL staining of liver sections n = 4–5 (**c**) Serum levels of TNF-α and IFN-γ in AhRR^+/+^, AhRR^E/+^, and AhRR^E/E^ mice after LPS injection (n = 5–6). (**d**) Systemic production of IL-12p70, IL-1β and IL-10 after LPS injection (n = 5–6). Expression of IL-1β and TNF-α in lung **(e)** and liver **(f)** of AhRR^+/+^, AhRR^E/+^ and AhRR^E/E^ mice that were either untreated (0 h) or treated with LPS for 3 h (n = 5–6). Data are shown as mean ± s.e.m.; significance was determined by one way ANOVA corrected for multiple comparisons by the Sidak method *p < 0.05, **p < 0.01 (AhRR^E/+^ or AhRR^E/E^ vs WT).

Next, we addressed the question whether the AhRR indeed acts as a functional repressor of the AhR *in vivo*. For this purpose, we first analyzed the immune response to high dose LPS injection as a model of septic shock. AhR-deficient mice have previously been shown to be more susceptible to LPS shock in accordance with enhanced production of proinflammatory cytokines^22^,^23^. In contrast, we found that AhRR^E/E^ mice are protected from LPS shock compared to WT and AhRR^E/+^ mice in survival experiments (Fig. 4a). This protection is also evident from the fact that liver cell apoptosis identified by TUNEL staining is significantly lower in AhRR^E/E^ versus WT mice (Fig. 4b). In addition, we observed reduced serum levels of TNF and IFN-γ (Fig. 4c), IL-12p70, IL-1β and IL-10 (Fig. 4d), as well as reduced IL-1β and TNF production in lung and liver (Fig. 4e,f) of AhRR^E/E^ mice. In some cases, diminished cytokine production was also observed in AhRR^E/+^ mice after LPS stimulation, indicating a gene dosage effect. Thus, in the LPS shock model AhRR-deficient mice behaved contrary to AhR-deficient mice, indicating that in this experimental model the AhRR indeed antagonizes AhR function *in vivo*.

### High susceptibility of AhRR-deficient mice to DSS colitis

**Figure 5:**
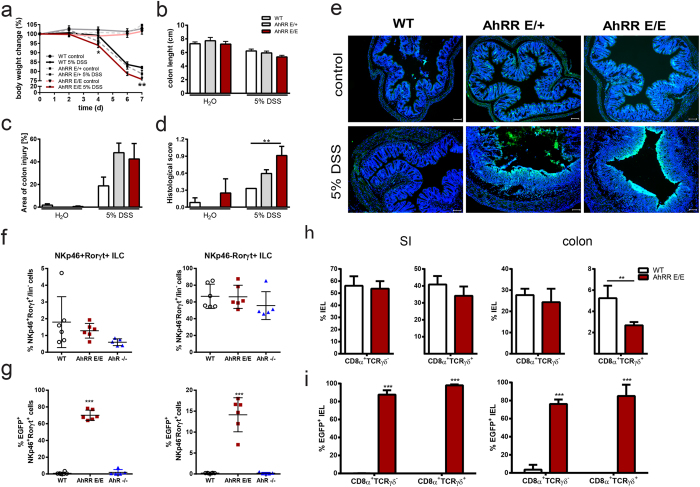
AhRR-deficiency aggravates symptoms of DSS induced colitis. **(a)** Colitis was induced in AhRR^+/+^, AhRR^E/+^ and AhRR^E/E^ mice by application of 5% DSS in the drinking water for 7d. Weight of mice was monitored every other day. *p < 0.05 and **p < 0.01 (AhRR^E/E^ vs WT) as determined by one way ANOVA corrected for multiple comparisons by the Sidak method. **(b)** Colon length at day 7 after DSS application (n = 7–11 mice). **(c)** The area of colon injury was calculated based on H&E stained histological sections (n = 4–5 mice). **(d)** The histological score was determined as described in Materials and Methods. Data are shown as mean ± s.e.m.; significance was determined by one way ANOVA corrected for multiple comparisons by the Sidak method **p < 0.01 (AhRR^E/E^ vs WT). **(e)** Immunofluorescence analysis of AhRR/EGFP expression and DAPI staining (blue) in colonic tissue after DSS application (d8) (bar: 100 μm). Data are representative for three independent experiments **(f)** Frequency of NKp46^+^ and NKp46^−^ RORγt^+^ ILC3 in the LP of the SI of WT, AhRR^E/E^ and AhR^−/−^ mice, n = 5–6. **(g)** Frequency of AhRR/EGFP expressing cells in the same subsets as shown in (**f**). Data are shown as mean ± s.d. **(h)** Frequency of CD8α^+^TCRγδ^−^ and CD8α^+^TCRγδ^+^ IEL in SI (left) and colon (right) (n = 3–6). **(i)** Frequency of AhRR/EGFP-expressing cells in WT and AhRR^E/E^ mice in the same IEL subsets as shown in **(h).** Data are shown as mean ± s.d. and significance was determined by one way ANOVA corrected for multiple comparisons by the Sidak method **p < 0.01, ***p < 0.001 (AhRR^E/E^ vs WT).

Absence of AhR signaling is also known to cause a severe dysregulation of lymphoid cell development and homeostasis in the gut^8^,^24^,^26^,^27^ as well as hypersensitivity to DSS-induced intestinal inflammation^24^,^25^. When subjected to DSS in the drinking water, AhRR^E/E^ mice, and to a lesser degree AhRR^E/+^ mice, were also highly susceptible to experimental colitis (Fig. 5). In light of the opposing effects of the AhR and AhRR in the LPS shock model, this result was not anticipated. AhRR^E/E^ and AhRR^E/+^ mice showed significantly enhanced weight loss over the 7 day experimental course (Fig. 5a) and a reduced colon length (Fig. 5b) compared with AhRR^+/+^ mice, although this difference was not statistically significant. Furthermore two of eleven AhRR^E/E^ DSS-treated mice died before the end of the experiment. In accordance, the infiltration of inflammatory cells in the colon as well as tissue damage was increased in AhRR^E/E^ and AhRR^E/+^ mice compared to WT mice (Supplementary Fig. 6 and Fig. 5c), resulting in a significantly enhanced overall disease score in AhRR^E/E^ and AhRR^E/+^ mice compared with control mice (Fig. 5d). Of note, heterozygous AhRR^E/+^ mice were also more strongly affected by DSS treatment than AhRR^+/+^ mice, pointing to a significant gene dosage effect when a functional AhRR is encoded from one allele only. Interestingly, at the end of the treatment period AhRR expression was strongly upregulated in the damaged intestinal mucosa (Fig. 5e).

The high susceptibility of AhR-deficient mice to intestinal inflammation has been attributed to a lack of IL-22 producing ILC3 and of intestinal epithelial lymphocytes (IEL)^8^,^24^,^26^,^27^. We therefore directly compared the frequency of these cell types in the intestinal mucosa of naïve WT, AhR^−/−^ and AhRR^E/E^ mice. Whereas NKp46^+^RORγt^+^ ILC3 (Fig. 5f left) and NKp46^−^RORγt^+^ ILC3 (Fig. 5f right) were slightly but not significantly reduced in AhR^−/−^ mice compared to WT mice, no such reduction was observed in AhRR^E/E^ mice. Expression of the AhRR/EGFP reporter was more abundant in the NKP46^+^ than in the NKp46^−^ ILC3 subset (Fig. 5g). As IL-22 producing ILC3 are known to be essential for protection against *Citrobacter rodentium* infection in the intestine^44^,^45^ we also tested resistance of AhRR^E/E^ mice to this bacterium in direct comparison to AhR^−/−^ mice and respective WT littermate controls. In agreement with published data, AhR^−/−^ mice were highly susceptible to *C. rodentium* infection with enhanced weight loss and lethality (Supplementary Fig. 7a). AhRR^E/E^ mice, in contrast, were as resistant to *C. rodentium* as their WT littermates, indicating normal functionality of ILC3 (Supplementary Fig. 7b). Whereas small intestinal and colonic CD8α^+^TCRγδ^−^ as well as CD8α^+^TCRγδ^+^ IEL were reduced to 60–90% in AhR^−/−^ mice (data not shown and^24^), their frequencies were normal in the small intestine of AhRR^E/E^ mice (Fig. 5h, left). In the colon, however, CD8α^+^TCRγδ^+^ IEL but not CD8α^+^TCRγδ^−^ IEL were also diminished by 50% in AhRR^E/E^ mice (Fig. 5h, right). Although the reduction in IEL in AhRR^E/E^ mice is much less pronounced than that seen in AhR^−/−^ mice, this partial deficiency may contribute to the enhanced susceptibility of AhRR^E/E^ mice to colitis. In agreement with published data on the expression of the AhR in ILC3^27^ and IEL^24^ the vast majority of both ILC3 and IEL strongly expressed the AhRR/EGFP reporter, indicating persistent activation of the AhR in these cell types (Fig. 5h,i). Taken together, AhRR-deficiency was found to cause enhanced susceptibility to DSS colitis similar to AhR-deficiency. In contrast to AhR^−/−^ mice, however, ILC3 were not affected by the absence of the AhRR, and only colonic CD8α^+^TCRγδ^+^ IEL were reduced by half in AhRR^E/E^ mice.

### Intestinal barrier integrity and composition of the microbiota

**Figure 6:**
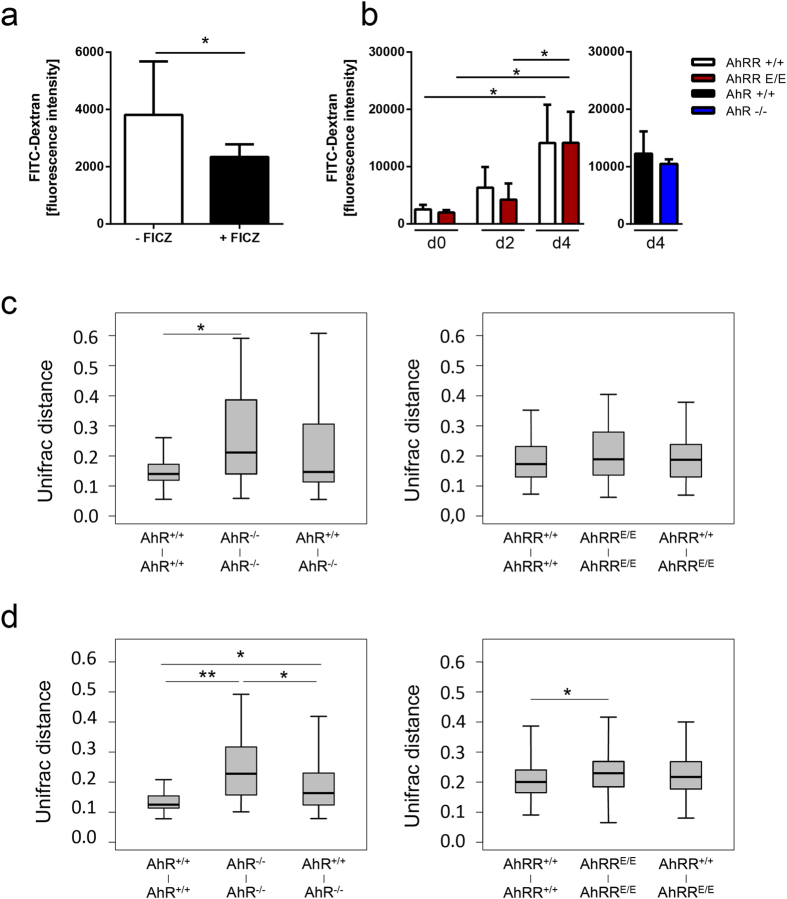
Intestinal permeability and composition of microbiota. **(a)** Dependency of intestinal permeability on AhR activation. WT mice were left untreated or fed with FICZ 16 h before oral treatment with FITC-Dextran. The fluorescence intensity of FITC-Dextran in serum was determined 4 h later (n = 7–8), Data are shown as mean ± s.e.m. and significance was determined by students *t*-test *p* < 0.05 **(b)** AhRR^E/E^ and AhRR^+/+^ littermates (left), were left untreated (d0, n = 3) or treated with 5% DSS for 2 (n = 3) or 4 days (n = 13, AhRR^+/+^ and AhRR^E/E^), and then fed with FITC-Dextran. AhR^−/−^ and AhR^+/+^ littermates (n = 3, right) were treated with 5% DSS for 4 days and then fed with FITC-Dextran. 4 h later fluorescence intensity of FITC-Dextran was measured in serum. Data are shown as mean ± s.e.m.; significance was determined by two way ANOVA corrected for multiple comparisons by the Sidak method *p < 0.05 (AhRR^E/E^ vs WT). **(c**,**d)** Variability of intestinal microbiota in the small intestine **(c)** and colon **(d)**. Stool samples were collected from the ileum and 16S rDNA was amplified from bacterial DNA and subsequently sequenced using the Ion Torrent PGM^TM^ system. Weighted unifrac distance analysis was performed using the operational taxonomic unit (OTU) information by FastUnifrac as described in Materials and Methods, n = 15 mice/group for AhRR^+/+^ and AhRR^E/E^ littermates and n = 8 for AhR^+/+^ and AhR^−/−^ littermates, Data are shown as mean ± s.d. and significance was determined by Wilcoxon’s rank sum test *p < 0,01 and **p < 1e-5.

As the frequency of IEL and the integrity of the intestinal barrier may significantly influence the susceptibility to colitis, we also assessed the permeability of the intestinal epithelium in WT, AhRR^E/E^ and AhR^−/−^ mice in steady state and at different time points after DSS treatment by oral application of FITC-Dextran. Oral application of the AhR ligand FICZ at steady state reduced the transmission of FITC-Dextran into the circulation (Fig. 6a). In contrast, DSS treatment led to an increase in mucosal permeability as expected but there were no differences between the genotypes (Fig. 6b). Thus, based on the uptake of FITC-Dextran we did not observe an increased permeability of the intestinal epithelium in the absence of either the AhR or AhRR, although treatment with FICZ strengthened the epithelial barrier. As another indicator of intestinal barrier dysfunction, we next assessed the composition of intestinal microbiota in naïve mice by next generation sequencing of bacterial 16S rDNA genes from small intestinal and colonic DNA samples of AhRR^+/+^ and AhRR^E/E^ littermates (15 mice/group), as well as AhR^+/+^ and AhR^−/−^ littermates (8 mice/group). In line with previous reports on a disturbed microbiome of AhR-deficient mice^8^,^24^ there was a much higher variability in microbial diversity between individual AhR^−/−^ mice than between their WT littermate controls in both small intestine (Fig. 6c, left) and colon (Fig. 6d, left) as determined by UniFrac distance analysis. In contrast, AhRR^E/E^ mice did not show differences in the diversity of small intestinal microbiota (Fig. 6c, right) and only a minor enhancement of microbial diversity in the colon (Fig. 6d, right). Therefore, there is no evidence that changes in the composition of intestinal microbiota explain the enhanced susceptibility of AhRR^E/E^ mice to DSS colitis.

### Enhanced Th17 and Tc17 differentiation in AhRR^E/E^ mice after induction of colitis

**Figure 7:**
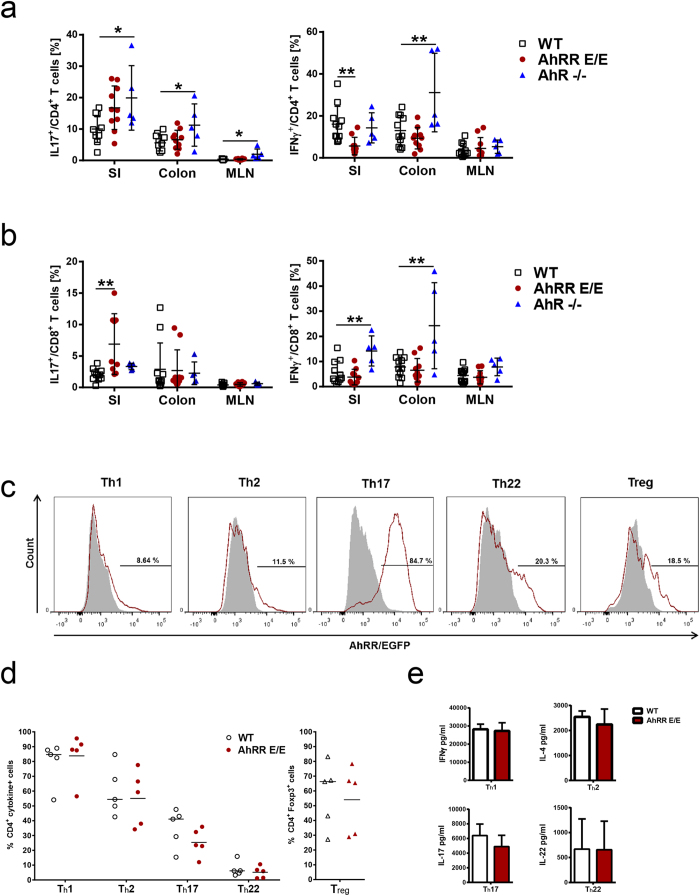
Influence of AhRR or AhR deficiency on T cell differentiation. **(a)** Frequency of IL-17A- or IFN-γ-producing CD4^+^ T cells in the SI and colonic lamina propria, and in MLN of WT, AhRR^E/E^ and AhR^−/−^ mice was determined by intracellular cytokine staining on d6 of DSS treatment, n = 5–11. **(b)** Frequency of IL-17A- or IFN-γ-producing CD8^+^ T cells in the SI and colonic lamina propria, and in MLN of WT, AhRR^E/E^ and AhR^−/−^ mice was determined by intracellular cytokine staining on d6 of DSS treatment, (n = 5–11). Data are shown as mean ± s.d. and significance was determined by one way ANOVA corrected for multiple comparisons by the Sidak method *p < 0.05 **p < 0.01 (AhRR^E/E^ vs WT or AhR^−/−^ vs WT) **(c)** Representative analysis of AhRR/EGFP expression of *in vitro* differentiated naïve splenic CD4^+^ T cells from WT (grey shaded histogram) and AhRR^E/E^ mice (red histogram) stimulated with anti-CD3 and anti-CD28 in the presence of specific cytokine cocktails and stimulated with PMA and Ionomycin as described in Materials and Methods. The fraction of EGFP^+^ cells in Th1, Th2, Th17, Th22, and Treg cell cultures is indicated. The data are representative of a total of 5 independent experiments. **(d)** Quantification of the proportion of IFN-γ (Th1), IL-4 (Th2), IL-17A (Th17) and IL-22 (Th22) producing CD4^+^ T cells, and the proportion of FoxP3^+^CD4^+^ T cells from cultures of WT and AhRR^E/E^ splenic T cells. **(e)** The concentration of the indicated cytokines in the different cultures as in **(c**,**d)** was determined by ELISA (n = 5). Data are shown as mean ± s.d.

Because AhR activation is known to influence the balance between Th17 and Treg cell differentiation in inflammation^9^,^11^, we next looked for differences in Treg representation and quantified relevant T effector cell subsets. First, the presence of FoxP3^+^CD4^+^ Treg was determined in the LP of the SI and colon as well as PP and MLN at steady state and at d6 of DSS treatment by FACS. The gating strategy is depicted in Supplementary Fig. 8a. While the frequency of FoxP3^+^CD4^+^ Treg cells slightly increased after DSS treatment, we could not detect any significant differences in Treg frequencies in WT and AhRR^E/E^ mice when analyzing either naïve or DSS-treated animals (Supplementary Fig. 8b,c). As for ILC and IEL, most Treg in the SI and about 40% of Treg cells in the colon expressed the AhRR/EGFP reporter at steady state, with the proportion of EGFP^+^ cells being somewhat lower after DSS treatment (Supplementary Fig. 8d,e). Second, the proportion of IL-17A- and IFN-γ-producing cells was measured after 6 days of DSS treatment within the CD4^+^ and CD8^+^ T cell compartment of the small intestinal and colonic LP as well as MLN. In both AhRR^E/E^ and AhR^−/−^ mice the frequency of Th17 cells was clearly enhanced over that of WT mice in the SI with a mean of 17% and 20% of CD4^+^ LP T cells in AhRR^E/E^ and AhR^−/−^ mice, respectively, compared with 10% in WT mice. In the colon, elevated frequencies of Th17 cells were only detected in AhR^−/−^ mice (Fig. 7a). These mice also possessed significantly increased proportions of IFN-γ producing Th1 and Tc1 cells in colon, and also had more Tc1 cells in the SI (Fig. 7a,b). In contrast, in AhRR^E/E^ mice Tc1 levels were normal, and Th1 differentiation was found to be strongly suppressed (Fig. 7a,b). Of note, significantly enhanced frequencies of Tc17 cells were observed in the SI of AhRR^E/E^ mice (mean of 7%) and to a lower extent in AhR^−/−^ mice (mean of 3%) (Fig. 7b). These findings indicate that effector T cell differentiation in the context of colitis is dysbalanced in both AhRR^E/E^ and AhR^−/−^ mice but with differential changes in Th1/Th17 and Tc1/Tc17 ratios.

To assess whether these differences are due to T cell intrinsic actions of the AhRR naïve T cells from the spleen of WT and AhRR^E/E^ mice were differentiated *in vitro* into Th1, Th2, Th17, Th22 or Treg effector cells. Whereas only very few Th1 and Th2 cells were found to be AhRR/EGFP^+^, more than 80% of Th17 cells and about 20% of Th22 and Treg cells expressed AhRR/EGFP in cultures of AhRR^E/E^ cells (Fig. 7c), in line with previous reports on preferential upregulation of the AhR in Th17 cells^11^,^13^,^16^. We then determined the proportion of cells producing the key cytokines IFN-γ (Th1), IL-4 (Th2), IL-17A (Th17) and IL-22 (Th22) by intracellular cytokine staining and ELISA (Fig. 7d,e). Treg cells were identified by expression of FoxP3. There were no differences in cytokine production nor frequency of FoxP3^+^ Treg comparing WT and AhRR^E/E^ mice, except for a slight but not significant reduction of IL-17-producing cells in AhRR^E/E^ Th17 cultures. Therefore, it can be concluded that the enhanced differentiation of Th17 cells and reduced frequency of Th1 cells in AhRR^E/E^ mice observed in the DSS colitis model is likely not due to a T cell intrinsic change in Th17 and Th1 cell differentiation.

### Elevated production of IL-1β in the intestine

**Figure 8:**
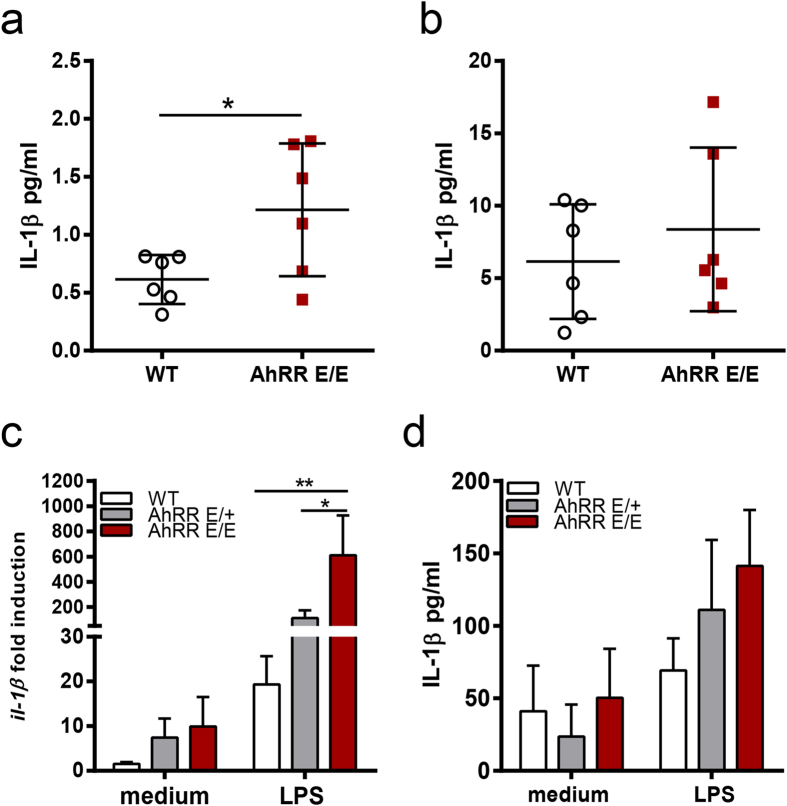
Enhanced expression of IL-1β in the intestine of AhRR-deficient mice. **(a)** colonic cultures of naïve WT and AhRR^E/E^ or **(b)** in mice fed for 6 days with 5% DSS were analyzed for expression of IL-1β (n = 6) Data are shown as mean ± s.d. and significance was determined by students t-test *p < 0.05 (AhRR^E/E^ vs WT). **(c)** pro**-***il-1*β was assessed by qPCR in BMMΦ of WT, AhRR^E/+^ and AhRR^E/E^ mice (n = 9–12). Data are shown as mean ± s.e.m. and significance was determined by one way ANOVA corrected for multiple comparisons by the Sidak method *p < 0.05 **p < 0.01 (AhRR^E/E^ vs WT or AhRR^E/E^ vs AhRR^E/+^). **(d)** IL-1β production in supernatants of BMMΦ of WT, AhRR^E/+^ and AhRR^E/E^ mice (n = 6). Data are shown as mean ± s.e.m.

A possible explanation for the differences in Th17/Tc17 differentiation *in vitro* and *in vivo* may be the local crosstalk of other AhRR-dependent cell types with T cells in the gut associated lymphoid tissues. Key cytokines of myeloid cells driving the differentiation of pathogenic IL-17 producing T cells are IL-23 and IL-1β 46,47. We therefore quantified the production of these cytokines in colon cultures of untreated and DSS-treated mice. Whereas IL-23 could not be detected in this set-up, we found that production of IL-1β was significantly enhanced in untreated AhRR^E/E^ mice compared with WT mice (Fig. 8a). At d6 after DSS treatment IL-1β production was about 10fold higher in both WT and AhRR^E/E^ mice (Fig. 8b). At this timepoint, we detected a significantly increased frequency of CD11c^+^MHCII^+^CD64^−^ DC in the SI of AhRR^E/E^ mice, whereas these DC were reduced in the MLN (Supplementary Fig. 9a). Within the DC population, a reduction was also seen for the CD103^+^CD11b^+^ DC subset in AhRR^E/E^ MLN, and an increased representation was observed for CD103^+^CD11b^+^-, CD103^+^CD11b^−^-, as well as CD103^−^CD11b^+^-DC subsets in PP (Supplementary Fig. 9b). There was no difference, however, in the frequency of F4/80^+^CD64^+^ macrophages comparing WT and AhRR^E/E^ mice (Supplementary Fig. 9c). We also directly determined the production of IL-1β by bone marrow-derived macrophages (BMMΦ). Remarkably, transcription of pro-IL-1β was strongly upregulated in the absence of the AhRR (Fig. 8c), and slightly elevated levels of secreted IL-1β could also be detected in cultures of AhRR-deficient BMMΦ although this difference was not significant (Fig. 8d). Therefore, increased levels of IL-1β may account for the enhanced Th17/Tc17 differentiation observed in the intestine of AhRR-deficient mice.

## Discussion

The AhR acts as a pattern recognition receptor for environmental aromatic chemicals, including anthropogenic and natural compounds. AhR-dependent upregulation of AhRR expression was suggested as part of a feedback control loop of AhR activity in the context of xenobiotic metabolism^36^,^48^, but little has been known so far about the role of the AhRR in immune regulation. Using a novel AhRR reporter and knockout mouse strain we here show that the AhRR indeed has a major impact on regulation of inflammatory immune responses. Surprisingly, the interplay between AhR and AhRR function within the immune system was found to be more complex than expected. In case of LPS-induced septic shock, genetic absence of the AhRR confers enhanced resistance, which is opposite to the high LPS sensitivity of AhR-deficient mice. On the other hand, when assessing its role in a gut-associated inflammatory response, AhRR deficiency aggravated symptoms of colitis similar to deficiency of the AhR itself. We here propose that this discrepancy depends on the restricted tissue- and cell type-specific expression of the AhRR, which is different from that of the AhR.

One of the most intriguing novel findings of this analysis is that expression of the AhRR in naive mice is fairly restricted to immune cells and is most prominent in or near the cutaneous and intestinal barrier. The highest frequency of AhRR expressing cells was detected in the small intestine, including CD11c^+^ myeloid cells, T cells, ILC3 and IEL. In line with *ahrr* being a target gene of the AhR, expression of the AhRR/EGFP reporter was apparently dependent on AhR activation in most cell types, with the exception of a subset of intestinal myeloid cells. Remarkably, the AhRR is only barely expressed in liver (Supplementary Fig. 1 and^40^), in which high activity of the AhR is crucial for xenobiotic metabolism^49^,^50^. These findings support the notion that regulation of AhR activation by AhRR occurs in a cell- and tissue-specific manner. Similarly, we could not detect AhRR in intestinal epithelial cells (IEC), in which expression of other AhR target genes such as Cyp1 genes is readily inducible^51^,^52^. In fact, a reciprocal activity of AhRR and Cyp1A1 has long been postulated for fibroblasts^53^,^54^,^55^,^56^. It is tempting to speculate that expression of the AhRR may restrain overwhelming AhR activation in immune cells of barrier organs, where AhR ligands like tryptophan photoproducts, nutritional or microbial components lead to sustained AhR activation. In contrast, in liver and the intestinal epithelium, where AhR activation is needed for metabolism of aromatic food constituents and detoxification of environmental pollutants, repression of AhR activation by AhRR might be deleterious. Thus, AhRR expression appears to be avoided in cells that are mainly involved in xenobiotic metabolism, whereas it plays a crucial role in the regulation of immune cell differentiation and function. This finding is further supported by the fact that a notable proportion of intestinal and cutaneous immune cells express AhRR even in AhR-deficient mice (Fig. 3e,f).

Our finding that genetic deficiency of either AhR or AhRR exacerbates intestinal inflammation in the DSS colitis model at first glance questions the inhibitory action of the AhRR on AhR activity. In light of the tightly controlled cell-type specific expression of the AhR and its target genes, one alternative explanation for this effect may be the differential importance of AhR versus AhRR in IEC versus immune cells of the LP. Recent work using conditional AhR-deficient mice demonstrated that the enhanced susceptibility to DSS colitis is seen only in IEC-specific AhR knockout mice, whereas T cell-specific ablation of AhR ameliorated colitis symptoms^57^. Thus, AhR deficiency in IEC may primarily enforce disruption of the epithelial barrier with a subsequent inflammatory response in the LP, whereas AhRR deficiency preferentially affects intestinal immune cells but not IEC as these cells do not appear to express *ahrr,* even in the WT background. It was previously shown that AhR deficiency leads to a substantial loss of IEL^24^, which significantly impairs the integrity of the epithelial barrier. Although the impact of AhRR deficiency on IEL was more subtle, we also detected a 50% reduction of colonic TCRγδ^+^ IEL in AhRR^E/E^ mice, which may contribute to the enhanced susceptibility of these mice to development of colitis.

Interestingly, we observed distinct differences in the frequencies of effector T cell subsets after DSS treatment in AhR- versus AhRR-deficient mice. In AhR^−/−^ mice, the frequency of Th17, Th1, as well as Tc1 cells but not Tc17 cells was enhanced, in line with previous findings in a different colitis model^8^. These authors proposed that the deficiency in ILC3 in AhR^−/−^ mice caused alterations in the microbiota such that Th17 cell differentiation was promoted. Other studies also reported a reduction in type 1 regulatory T (Tr1) cell function in AhR^−/−^ mice, affecting c-Maf-dependent IL-10 expression^21^ and expression of miR132/212^15^,^57^. Dioxin treatment on the other hand caused protection from DSS colitis accompanied by elevated frequencies of FoxP3^+^ Treg and PGE_2_ production^9^,^19^. We did not observe alterations in FoxP3^+^ Treg cell frequencies in AhRR^E/E^ mice either in steady state or after DSS treatment, and ILC3 functions were also found to be intact as judged by the normal resistance of the mice to *C. rodentium* infection (Supplementary Fig. 7b). Instead, absence of the AhRR mainly caused enhanced differentiation of Th17 and Tc17 cells accompanied by a relative reduction of Th1 and Tc1 cells. It has recently been demonstrated that *ahr* is a target gene of RORγt^58^. Thus, assuming the constitutive presence of AhR ligands in the gut, RORγt is likely to indirectly also enhance *ahrr* transcription in Th17 cells. In *in vitro* culture systems AhR ligands enhance Th17 cell differentiation and IL-17 production^6^,^11^,^14^,^15^,^16^,^17^ which would be further boosted in the absence of AhRR-dependent feedback inhibition. As shown in Fig. 7d,e, however, we did not observe enhanced Th17 differentiation of AhRR-deficient splenic T cells *in vitro*, although these cells strongly expressed the AhRR/EGFP reporter. A possible difference of these cultures to the *in vivo* environment is that they are independent of myeloid cell-derived cytokines driving Th17/Tc17 differentiation. Indeed, we could show that AhRR-deficient mice have elevated levels of intestinal IL-1β already at steady state, and that pro-IL-1ß transcripts as well as the bioactive form of IL-1β are upregulated in the absence of AhRR expression. As AhR activation was shown to upregulate IL-1β production^59^, our findings are in agreement with an enhanced AhR activity in myeloid cells lacking AhRR-mediated feedback inhibition. In addition, the frequency of DC but not macrophages appeared to be increased in the SI of DSS-treated AhRR^E/E^ mice. Curiously, in the LPS shock model we found a strong reduction of IL-1β in the liver of AhRR^E/E^ mice. It is intriguing in this context that AhRR expression is not detectable in adult liver, whereas the intestine is the organ with the highest expression levels of AhRR. One may speculate that AhRR expression in fetal liver^43^ induces epigenetic changes in liver resident macrophages, which may specifically alter their function throughout adulthood different from intestinal macrophages, which have a high rate of turn-over.

Another interesting finding is the enhanced Tc17 frequency, which we observed in AhRR^E/E^ but not AhR^−/−^ mice after DSS treatment. Tc17 cells have a reduced cytotoxic activity compared to classical Tc1 cells but similar to Th17 cells produce IL-17 and IL-21 (for review see^60^). These cells have been associated with autoimmune diseases and were shown to promote intestinal inflammation in a RAG transfer colitis model^61^. Therefore, the enhanced susceptibility to DSS colitis in AhRR^E/E^ mice may be caused by the elevated frequency of Th17 as well as Tc17 cells in agreement with the pathogenic role of IL-17A production in the DSS colitis model^62^. In contrast, IFN-γ producing T cells were nearly absent in DSS-treated AhRR^E/E^ mice, indicating a substantial impact of the AhRR on IFN-γ expression. In light of the observed enhancement of IFN-γ production in AhR^−/−^ mice (Fig. 7 and^63^), this finding can be explained by a direct inhibition of AhR activity by AhRR despite the enhanced susceptibility of both AhR- and AhRR-deficient mice to DSS colitis. As IFN-γ is known to inhibit Th17 differentiation through activation of Stat1 and AhR causes accelerated dephosphorylation of Stat1 in T cells^16^, the absence of IFN-γ dependent Stat1 activation may further facilitate Th17/Tc17 differentiation in AhRR^E/E^ mice.

Besides adaptive immunity, the AhR was shown to also play an important role in innate immunity. The AhR is able to interact with the NF-κB family members RelA and RelB^33^. Furthermore, the AhR negatively regulates the proinflammatory response to LPS in macrophages in a Stat1- and NF-κB-dependent manner^22^. AhR-deficient mice are highly susceptible to LPS induced septic shock, probably as a result of enhanced proinflammatory cytokine production by AhR-deficient macrophages^22^,^23^. In addition, enhanced AhR activation by application of 3MC prior to LPS shock enhances survival^23^. In line with these data, we could demonstrate that deficiency of the AhRR confers protection against LPS-induced shock. Endotoxin shock is mainly mediated by pro-inflammatory cytokines produced by immune cells such as macrophages. As discussed above, in DSS colitis, also non-hematopoetic cell populations, like enterocytes, are involved in the immunopathology of the disease. Differential, non-overlapping expression profiles of the AhRR and AhR-dependent xenobiotic metabolizing enzymes, which are all encoded by target genes of the AhR, in certain cell populations might therefore explain the specific phenotypes of AhR- or AhRR-deficient mice. Overall, we think that most of the phenotypic changes in AhRR-deficient mice described here are still compatible with an inhibitory function of the AhRR on the AhR, considering the selective expression pattern of the AhRR. In conclusion, our data provide first evidence that microbial, nutritional or environmental stimuli contribute to AhRR expression in immune cells of barrier organs like skin and gut as a result of continuous AhR stimulation. In this way, AhR activity appears to be locally controlled by feedback inhibition through the AhRR in a cell type-specific manner.

## Experimental Procedures

### Targeting the murine *ahrr* locus by homologous recombination

The targeting vector was constructed such that an EGFP cDNA together with a polyA signal and a loxP-flanked neomycin resistance cassette was inserted into the second exon of the *ahrr*. To avoid transcription of a truncated protein by alternative splicing, the third exon was deleted additionally. Homologously recombined ES cell clones (E14K) were detected by Southern blot hybridization after digestion of ES cell DNA with DraI and hybridized to a 5′flanking probe (Supplementary Fig. 1a) yielding a 6.6 kb fragment for the WT allele and a 3.6 kb fragment for the mutated allele. Germline transmission of the targeted allele was confirmed by Southern blot analysis. The neomycin resistance cassette was removed from the targeted allele by crossing mutant mice with a Cre-Deleter-strain (CD11cCre3 mice; IF, unpublished). In this study, heterozygous AhRR^E/+^ reporter mice and homozygous AhRR^E/E^ mice expressing EGFP but not AhRR were used. WT littermates were used as controls. Mice were originally generated on a mixed C57BL/6J/129Ola genetic background and backcrossed to C57BL/6J for 2–9 generations. Primers for typing of mice were as follows: AhRR-rev:3′-tccttctcttcctaccggcg-5′; AhRR-fwd: 3′-catagtggaagtccagcacataga-5′; AhRR/GFP: 3-tccttgaagtcgatgccctt-5′ (Supplementary Fig. 1C). Because the AhR of 129Ola mice has a low affinity to certain AhR-ligands, mice were screened for expression of the high affinity C57BL/6 allele by PCR as described^40^. In all experiments mice with this high affinity allele of the AhR were used.

AhR^−/−^ mice^64^ were bred in our animal facility. To analyze the contribution of AhR activation to AhRR-expression we generated AhR^−/−^AhRR^E/+^ mice by intercrossing AhR^−/−^ mice to AhRR^E/E^ mice. Male and female mice aged between 6–12 weeks were used for the experiments and were bred according to German guidelines for animal care. All experiments were performed according to German and Institutional guidelines for animal experimentation and were approved by the government of North Rhine**-**Westphalia (Germany).

### TLR or AhR stimulation *in vivo*

Mice were injected i.p. with *E. coli* 0111:B4 LPS (15 mg/kg in 200 μl) in PBS or with 3MC (25 mg/ml, both Sigma, Deisenhofen, Germany) in PBS/1% DMSO for 16 h or treated per gavage with 250 μg 3MC in DMSO/olive oil (1:4 v/v). Control animals received the respective solvent.

### Immunohistology

Tissue samples were fixed for 3 h in 4% paraformaldehyde (PFA) at 4 °C and saturated in a sucrose gradient from 5% to 20% sucrose. Frozen sections were counterstained with 0.5 μg/ml DAPI (4,6-diamidino-2-phenylindole). For some experiments slides were stained with antibodies against B220 (RA3-6B2, eBioscience, San Diego, CA USA) or EpCAM (G8.8 Biolegend). Images were acquired with a Keyence B2900 digital microscope (Keyence Corporation, Osaka, Japan) and analyzed with BZII Analyzer software (Keyence Cooperation).

### Flow cytometry

For preparation of skin cell suspensions from dorsal skin subdermal fat was removed, the skin explants were incubated for 2 h in PBS/0.25% trypsin, 5 mM EDTA (Invitrogen, Karlsruhe, Germany) at 37 °C to separate the epidermis from the dermis. Epidermal sheets were mechanically disrupted to obtain a single cell suspension. Dermal sheets were cut into small pieces and incubated for 2.5 h in collagenase D (1.6 mg/ml, activity: 226 U/mg; Roche, Mannheim, Germany) and thereafter mechanically disrupted. Cell suspensions were stained with antibodies against MHCII (clone M5/114.15.2) and CD24a (M1/69, both eBioscience). For preparation of IEL and LPL the small intestine was flushed with PBS and PP were removed. The tissue was cut into pieces and incubated in 15 mM HEPES, 5 mM EDTA, 10% FCS in PBS for 45 min at 37 °C and filtered through a 70 μM cell strainer. For LPL isolation mucus removal of intestinal tissue pieces was performed in 5 mM DTT, 2% FCS, 100 U/ml Penicillin and 100 ug/ml Streptomycin in HBSS for 20 min at 37 °C. Epithelial cells were removed by incubation in 5 mM EDTA, 2% FCS, 100 U/ml Penicillin and 100 μg/ml Streptomycin in HBSS for three times 15 min at 37 °C and washed with 10 mM Hepes, 100 U/ml Penicillin and 100 μg/ml Streptomycin in HBSS for 10 min at 37 °C. Tissue was digested with 4 U/ml Liberase and 4000 U/ml DNaseI in 10 mM Hepes, 100 U/ml Penicillin and 100 ug/ml Streptomycin in HBSS for 45 min at 37 °C and afterwards filtered through a 70 μm cell strainer. For preparation of PP, LN and splenic cell suspensions, tissues were meshed through a 100 and 70 μm cell strainer. Cell suspensions were stained with antibodies against CD3 (145-2C11), CD4 (RM4-5), CD8 (53-6.7), CD19 (MB19-1), CD25 (PC61, BD Biosciences, Heidelberg, Germany, MHCII (M5/114.15.2), CD11c (HL-3), F4/80 (CI:A3-1, Abd Serotec, Oxford, UK), CD11b (M1/70), NK1.1 (PK136), CD103 (2E7), CD64 (X54-5/7.1),TCRγδ (GL3), Foxp3 (FJK16s), IFN-γ (XMG1.2), IL-17 (eBio17B7), NKp46 (29A1.4) and RORγt (AFKJ-9). If not indicated otherwise, antibodies were purchased from eBioscience. For intracellular cytokine and transcription factor analysis cells were fixed with 2% paraformaldehyde for 20 min, permeabilized with 0.5% Saponin in PBS/BSA and stained for 60 min at RT in the dark. For anti GFP staining incubation was performed overnight at 4 °C. Cell populations were analyzed with a FACScalibur flow cytometer and a LSRII Cytometer (BD Biosciences); data were analyzed with FlowJo software (Tree star, Ashland, USA).

### Generation of BMDC and BMMΦ

For generation of BMDC and BMMΦ bone marrow cells were differentiated in medium supplemented with 2% supernatant of GM-CSF-transfected X63Ag8-653 cells or 10% supernatant of L929 cells. For expression analysis cells were either stimulated with LPS (1 μg/ml) or 3MC (10 μM; Sigma Darmstadt, Germany) for 16 h. For cytokine measurements supernatants were analyzed by ELISA for production of IL-1β (R&D Systems, Wiesbaden, Germany).

### Real-time PCR-analysis

BMDC and BMMΦ were stimulated with LPS (1 μg/ml) or 3MC (10 μM) for 3 h. RNA extractions were carried out using the RNeasy Fibrous tissue mini kit (Qiagen, Hilden, Germany). First-strand cDNA was synthesized from 1 μg of total RNA using Revert Aid reverse transcriptase (Thermo Fisher Scientific, Bonn, Germany). Real time PCR was performed on an SDS7300 cycler (Applied Biosystems, Foster City, CA) using absolute SYBR-green ROX master mix (Thermo Fisher Scientific). Primers were designed using the Universal Probe Library (Roche Applied Science, Mannheim, Germany), primers for *cyp1A1* were described in^65^, primers for AhRR were described in^54^. Primers for EGFP and IL-1β were: EGFPfwd: 5′-aagggcgaggagctgttcac-3′ and EGFPrev: 5′-ttgtgccccaggatgttgcc-3′; IL-1βfwd: ttgacggaccccaaaagat and IL-1βrev: gaagctggatgctctcatca.

### Induction of LPS shock

Age-matched male AhRR^+/+^, AhRR^E/+^ and AhRR^E/E^ mice were used for LPS challenge at an age of 6–10 wk. 15 mg/kg LPS (Sigma) was diluted in sterile PBS and injected i.p. Survival was monitored over 8d. Peripheral blood, spleen, liver and lung were collected before, 1.5 and 3 h after LPS injection. Organs were snap-frozen in liquid nitrogen and homogenized after thawing in 1 ml PBS containing complete 10% protease inhibitors (Roche). Cytokine concentrations in serum and organ extracts were measured by ELISA (R&D Systems) and were normalized against the protein concentration in each lysate.

### TdT-mediated dUTP-biotin nick end labeling (TUNEL) staining

The amount of apoptotic cells in the liver of naïve and 16 h LPS-treated mice was calculated by TUNEL on tissue sections using the *in situ* cell death detection kit (Roche) according to the manufacturer’s instructions. After mounting, the number of TUNEL positive cells was counted at 200x magnification in 5–6 randomly chosen fields per liver using a Keyence B2900 digital microscope (Keyence Corporation) and analyzed with BZII Analyzer software (Keyence Cooperation).

### DSS colitis model

DSS colitis was induced in age matched female AhRR^+/+^, AhRR^E/+^ and AhRR^E/E^ mice by adding 5% DSS (w/vol) to the drinking water for 7d. Weight, stool consistency and rectal bleeding were assessed every other day. At day 7 colons were removed and colon length was measured. Frozen sections of the distal colon were analyzed for infiltration of inflammatory cells. Paraffin sections of whole colon samples were stained with H&E. The sections were evaluated histopathologically in a blinded manner. Each colon sample was divided into three segments of identical length and each segment was scored individually. Shortly, the degree of infiltration of inflammatory cells and mucosal erosion/ulceration was graded from none (0) to mild (1), moderate (2) and severe (3). Additionally, the percentage of altered tissue was calculated visually for each segment of identical length. The mean value of the scores from each individual intestinal segment was calculated as the total score for each animal. For cytokine analysis, colonic segments were incubated in RPMI1640, 10% FCS for 6 h. IL-1β was measured in the supernatant by ELISA (R&D Systems) and was calculated as pg/ml per mg tissue.

### Determination of intestinal permeability

Intestinal permeability was assessed by the transduction of orally applied FITC-Dextran into the bloodstream. Mice were starved for 4 h and 600 mg/kg FITC-Dextran (MW 4000, Sigma) in PBS was applied. Blood was sampled 4 h later. 30 μl serum were analyzed in a Spectrophotometer (Tecan, Mennedorf, Switzerland) with an excitation wavelength of 492 nm and an emission wavelength of 525 nm.

### *In vitro* T cell differentiation

Naïve CD4^+^ splenic T cells were isolated using MACS Pan T cell Isolation Kit II and CD8a MicroBeads (both: Miltenyi, Bergisch Gladbach, Germany), followed by purification FACS sorting of CD4^+^CD44^lo^CD62L^hi^CD25^−^ cells using a FACSAriaII (BD Biosciences). 1×10^5^ cells were cultured in IMDM (Life Technologies GmbH, Darmstadt, Germany) and activated with plate-bound anti-CD3 (3 μg/ml, Biolegend) and soluble anti-CD28 (1 μg/ml, Biolegend) for 5 days at 37 °C. For maintaining T_h_0 cells, anti-IFNγ (10 μg/ml, Biolegend) and anti-IL-4 (10 μg/ml, Biolegend) were added. For differentiation to T_h_1 cells IL-12 (20 ng/ml) and anti-IL-4 (10 μg/ml), and to T_h_2 cells IL-4 (100 ng/ml) and anti-IFN-γ (10 μg/ml) were added. IL-6 (100 ng/ml), TGF-β (5 ng/ml), FICZ (300 nM, Enzo Life Sciences, Lörrach, Germany), anti-IL-4 (10 μg/ml) and anti-IFNγ (10 μg/ml) were used for differentiation to T_h_17 cells. For differentiation to T_h_22 cells IL-6 (40 ng/ml), IL-23 (20 ng/ml) and FICZ (300 nM), and for induction of regulatory T cells, TGF-β (5 ng/ml) and retinoic acid (2.5 ng/ml, Sigma) were added. All cytokines used were purchased from PeproTech, Rocky Hill, NJ, USA. Cytokine concentrations in cell supernatants were measured by ELISA for IFN-γ, IL-4, IL-22, TGF-ß (all R&D Systems) and IL-17 (Biolegend). For intracellular cytokine staining, cells were re-stimulated for 4 h with PMA (50 ng/ml, Sigma), ionomycin (1 μg/ml, Sigma) and monensin (2 μl/ml, Biolegend). After staining with CD4 (RM4-5) or CD4 and CD25 (PC61), cells were fixed and permeabilized to allow intracellular staining with antibodies for IFNγ (XMG1.2 BD), IL-4 (11B11); IL-17 (17B7) and IL-22 (1H8PWSR, all BD Pharmingen) overnight. *In vitro* differentiated T_reg_ cells were treated with FoxP3/Transcription Factor Staining Buffer Set (eBioscience) as suggested by the manufacturer and stained with FoxP3 (FJK-16s, eBioscience).

### Next generation sequencing and 16S rDNA sequence analysis

DNA was isolated from stool samples using the QIAamp DNA Stool Kit (Qiagen). Bacterial 16S rDNA genes of metagenomic DNA extracted from the SI and colon of 15 or 16 individual AhRR^+/+^ and AhRR^E/E^ littermates, respectively, and 8 individual AhR^+/+^ and AhR^−/−^ littermates were PCR amplified using modified 27F (5′-AKWGTTTGATCMTGGCTCAG) and 338R (5′-CTGCWGCCWYCCGTAGRWGT) eubacteria 16S rDNA universal primers conjugated with randomly selected Ion Xpress^TM^ barcode adapter sequences. PCR was conducted based on the protocol for the PrimeSTAR Max DNA Polymerase (TakaraBio, Shiga, Japan) with annealing temperature set at 47 °C and number of cycles at 25. Subsequent sample preparation was conducted based on the protocol for amplicon sequencing with the Ion Torrent PGM^TM^ system (Life Technologies Inc., Tokyo, Japan). Quality and concentration of all samples were determined using the 2100 Bioanalyzer (Agilent Technologies, Tokyo, Japan).

For sequence analysis, raw reads were first processed with the pipeline introduced by Kim *et al*.^66^ to trim the primer sequences and remove low quality (average quality score <25) reads. Next, reads shorter than 300 bp were excluded from subsequent analyses. Taxonomy of the reads was then annotated using RDP classifier^67^ based on the RDP v10 reference files. Here, the assigned bacterial taxonomies were accepted if their bootstrap value was over 50%. Reads showing low bootstrap values were classified as “undetermined” in the generated results. All the reads were clustered into operational taxonomic units (OTUs) with 97% identity by UPARSE^68^. To investigate the differences between the bacterial population of each mouse gut sample, we conducted a weighted Unifrac analysis using the OTU information by FastUnifrac^69^. For the Unifrac analysis, representative sequences from each OTU were aligned by clustal-omega^70^ and a phylogenetic tree was built using PHYLIP^71^ based on the neighbor-joining method. All sequence data have been deposited in the SRA database under the following accession number: SRP067599.

### Statistical analysis

Data were analyzed with Prism 6 (Graph Pad Software) using Student’s *t* test, one way and two way ANOVA with Sidaks posttest, and log rank test for survival curves as indicated in the text. The level of significance for p < 0.05 was denoted as (*) or, for  < 0,01 as (**), for p < 0,001 as (***) as indicated in the figure legends.

## Additional Information

**How to cite this article**: Brandstätter, O. *et al*. Balancing intestinal and systemic inflammation through cell type-specific expression of the aryl hydrocarbon receptor repressor. *Sci. Rep.* **6**, 26091; doi: 10.1038/srep26091 (2016).

## Supplementary Material

Supplementary Information
