# Supplementary material for: Balancing intestinal and systemic inflammation through cell type-specific expression of the aryl hydrocarbon receptor repressor
Source: Sci Rep. 2016 May 17;6:26091. doi: 10.1038/srep26091 (PMC4869119; doi:10.1038/srep26091)
Supplement: Supplementary Information [file srep26091-s1.pdf]

Supplementary Information

Balancing intestinal and systemic inflammation through cell type-specific expression of the aryl hydrocarbon receptor repressor

Olga Brandstätter[1,2,6], Oliver Schanz[1,6], Julia Vorac[1,2,6], Jessica König[1], Tetsushi Mori[3], Toru Maruyama[3], Markus Korkowski[2], Thomas Haarmann-Stemmann[2], Dorthe von Smolinski[4], Joachim L. Schultze[5], Josef Abel[2], Charlotte Esser[2], Haruko Takeyama[3], Heike Weighardt[1,2,7] and Irmgard Förster[1,2,7]

[1]Immunology and Environment, Life and Medical Sciences (LIMES) Institute, University of Bonn, Carl-Troll-Straße 31, 53115 Bonn, Germany
[2]IUF-Leibniz Research Institute for Environmental Medicine gGmbH, Auf´m Hennekamp 50, 40225 Düsseldorf, Germany
[3]Center for Advanced Biomedical Sciences (TWIns), Waseda University, 2-2, Wakamatsu-cho, Shinjuku-ku, 162-8480, Tokyo, Japan
[4]Institut für Tierpathologie der FU Berlin, Robert von Ostertag Strasse 15, 14163 Berlin
[5]Genomics and Immunoregulation, Life and Medical Sciences (LIMES) Institute, University of Bonn, Carl-Troll-Straße 31, 53115 Bonn, Germany
[6]these authors have equally contributed to this work
[7]joint senior authors

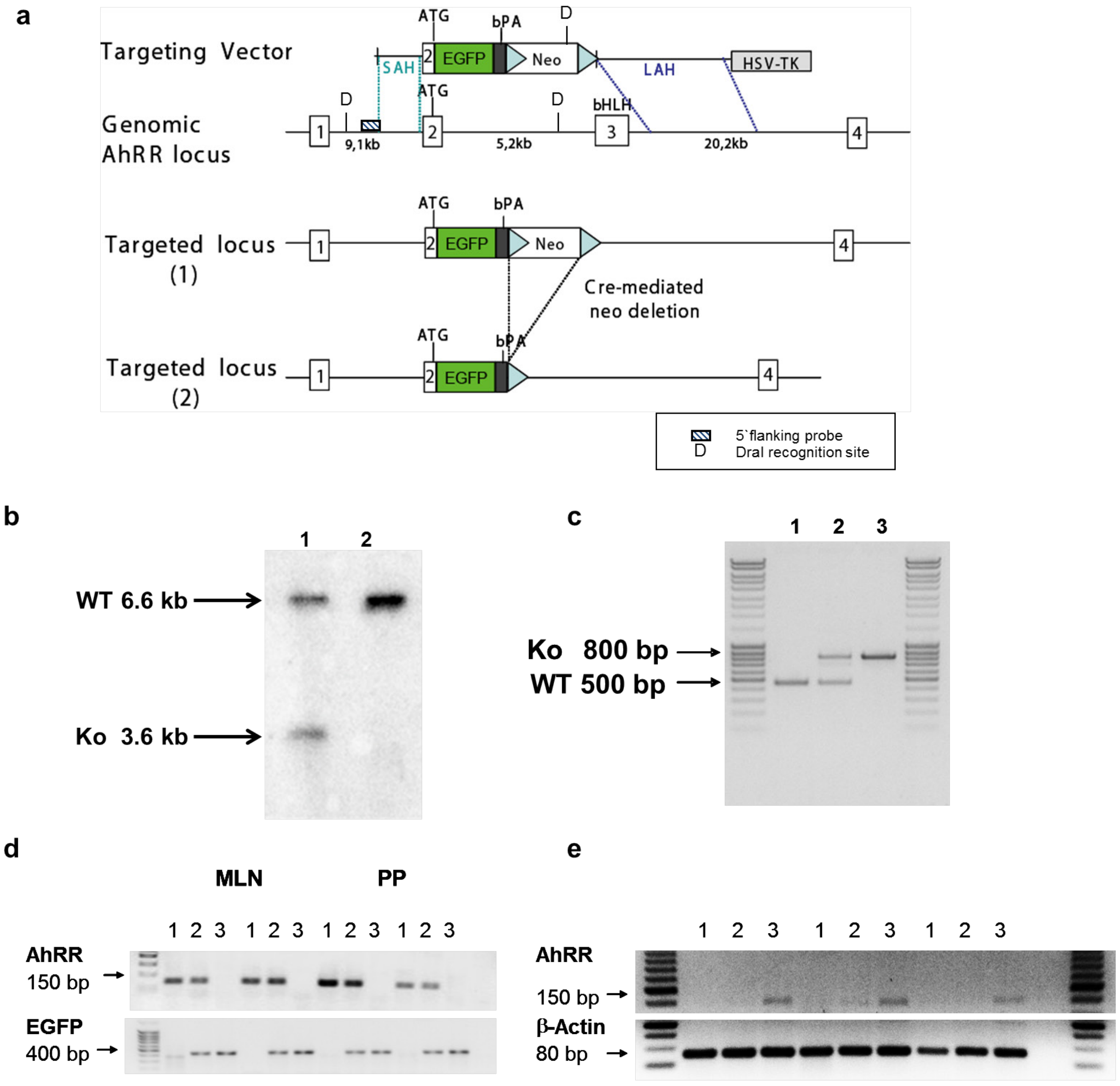

Supplementary Figure 1: **Generation of AhRR-deficient mice**

(a) Targeting-strategy to generate AhRR-deficient mice. The EGFP-cDNA was inserted into the second exon of AhRR and the third exon was deleted. In addition, a loxP-flanked neomycin gene was inserted. For Southern blot analysis a 5`flanking probe was used. (**b)** For Southern blot analysis DNA of ES cells was digested with Dra1. Hybridisation with a 5`-flanking probe revealed a 6.6 kb (WT) and a 3.6 kb (Ko) fragment in the targeted ES cell clone (1) compared to WT ES cells (2). **(c)** PCR of tail DNA of WT (1), AhRR$^{E/+}$ (2) and AhRR$^{E/E}$ (3) mice shows a 500 bp fragment for the WT allele and an 800bp fragment for the

mutant allele. **(d)** Expression analysis of endogenous *ahrr* and transgene *egfp* in MLN and PP of 2 independent WT (1), $AhRR^{E/+}$ (2) and $AhRR^{E/E}$ (3) mice by RT-PCR. (**e**) Expression analysis of endogenous *ahrr* in liver (1), lung (2) and MLN (3) of 3 independent WT mice by RT-PCR. β-Actin was used as loading control. The last lane contains a control without cDNA.

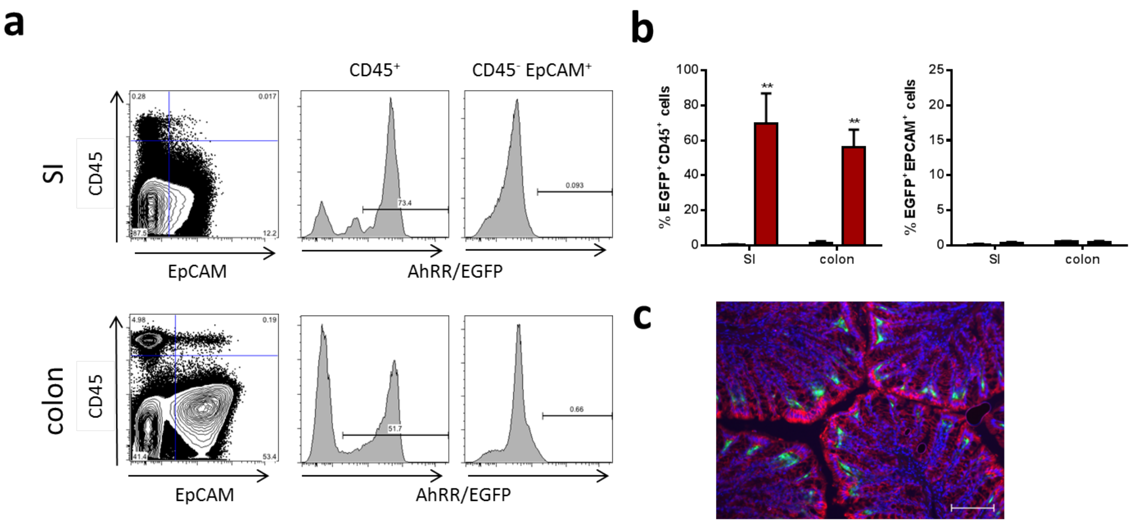

Supplementary Figure 2: No AhRR expression in intestinal epithelial cells

(a, b) Flow cytometry of single cell suspensions of the colon (n=2-4) of WT and AhRR$^{E/E}$ mice. Data are shown as mean ± s.d. and significance was determined by students *t*-test $^{**}p < 0.01$ (AhRR$^{E/+}$ vs WT). **(c)** Immunofluorescence analysis of frozen sections of the colon of AhRR$^{E/E}$ mice counterstained with EpCAM (red) to visualize intestinal epithelial cells and DAPI (bars: 100µm).

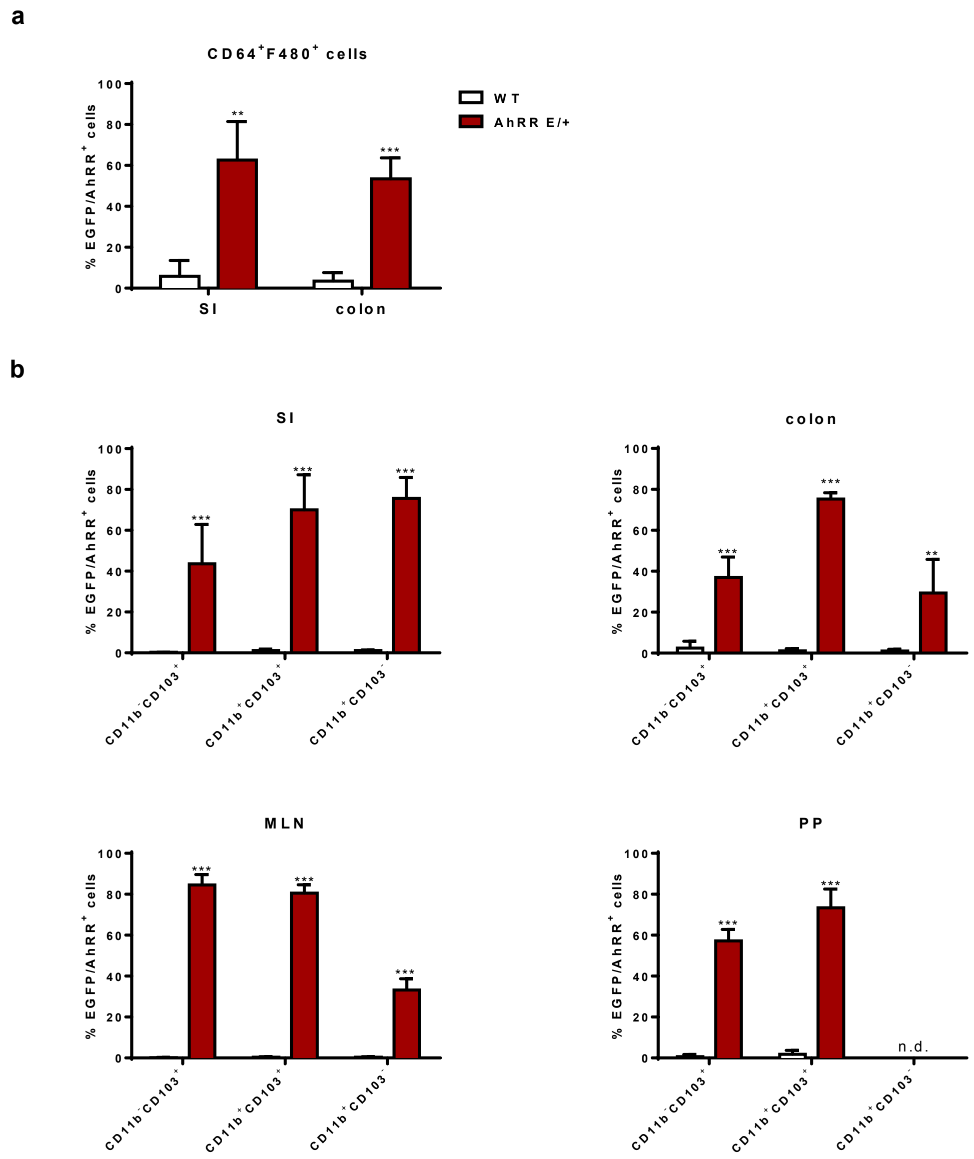

Supplementary Figure 3: Expression of the AhRR in myeloid cell populations

(a) Frequency of AhRR/EGFP expressing $CD64^{+}/F480^{+}$ Macrophages. **(b)** Frequencies of AhRR/EGFP expressing $CD11b^{-}CD103^{+}$, $CD11b^{+}CD103^{+}$ and $CD11b^{+}CD103^{-}$ DC of $CD11c^{+}MHCII^{+}$ cells in MLN, PP, SI and colon (n=6). Data are shown as mean ± s.d. and significance was determined by students *t*-test $^{**}p < 0.01$, $^{***}p < 0.001$ ($AhRR^{E/+}$ vs WT).

.

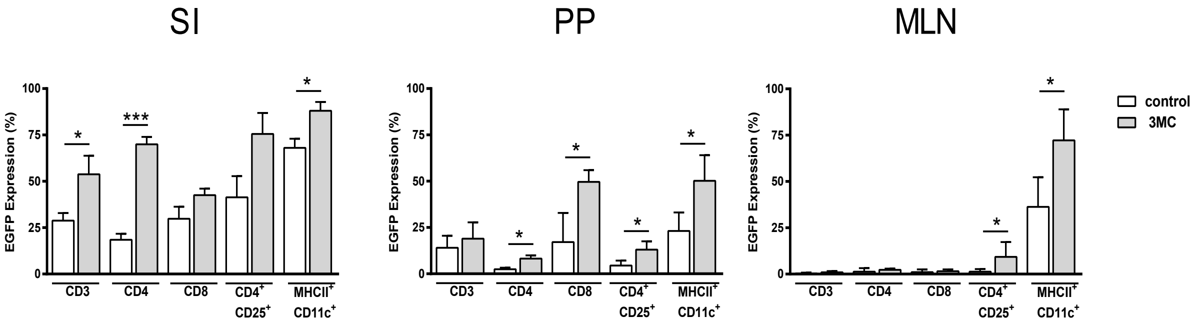

Supplementary Figure 4: 3MC treatment enhances AhRR expression in vivo

Frequencies of AhRR/EGFP^{+} cells in T cell and myeloid cell populations in SI, PP and MLN of AhRR$^{E/+}$ mice after treatment with 3MC or solvent p.o. (n= 3-5). Data are shown as mean ± s.d. and significance was determined by students *t*-test **p* <0.05, ***p* < 0.01, ***p< 0.001 (AhRR$^{E/+}$ controls vs AhRR$^{E/+}$ 3MC).

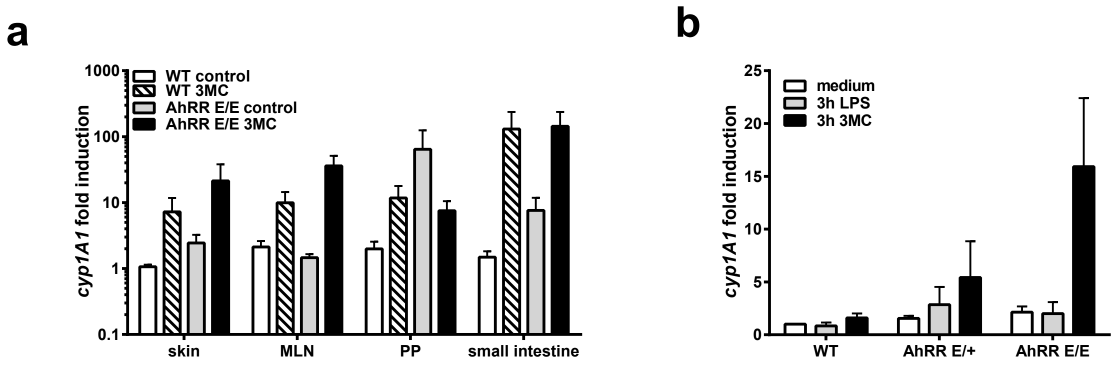

Supplementary Figure 5: Enhanced levels of *cyp1A1* in AhRR-deficient mice

(a) *cyp1A1* mRNA levels in skin, MLN, PP and small intestine LP from WT and AhRR$^{E/E}$ mice treated with 3MC in DMSO/olive oil (1:4 v/v) or with solvent per gavage (n= 4). **(b)** *cyp1A1* mRNA levels from BMDC of WT, AhRR$^{E/+}$ and AhRR$^{E/E}$ mice treated with LPS or 3MC for 3h (n=4 mice per group and time point).

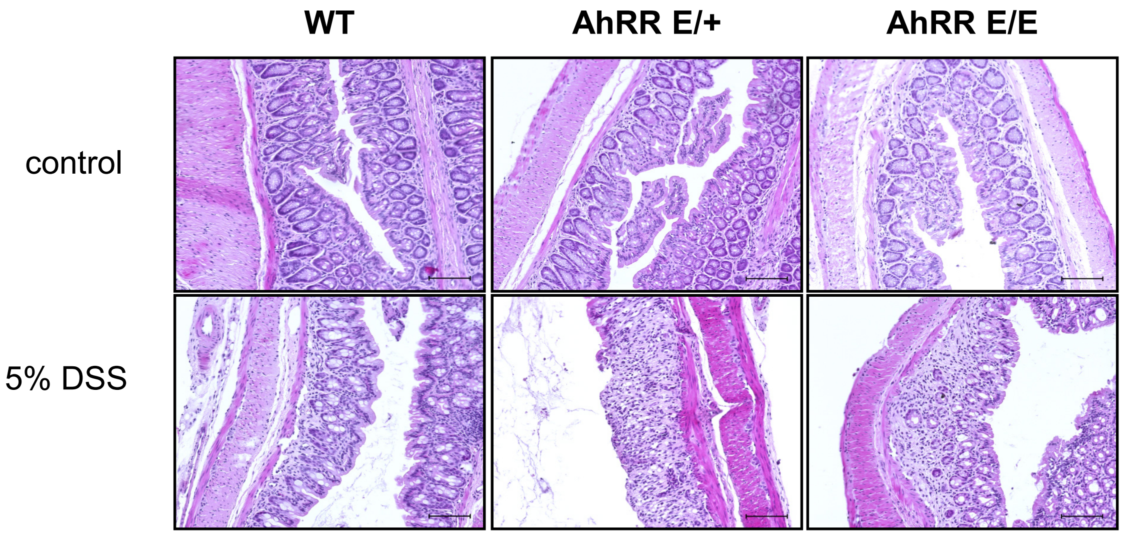

Supplementary Figure 6: Histologic analyses of colonic samples

Histologic analysis of colons of WT, $AhRR^{E/+}$ and $AhRR^{E/E}$ mice at d7 after colitis induction. H&E stained frozen sections of colons of control or DSS-treated $AhRR^{E/+}$ and $AhRR^{E/E}$ mice (one representative experiment of 3 is shown).

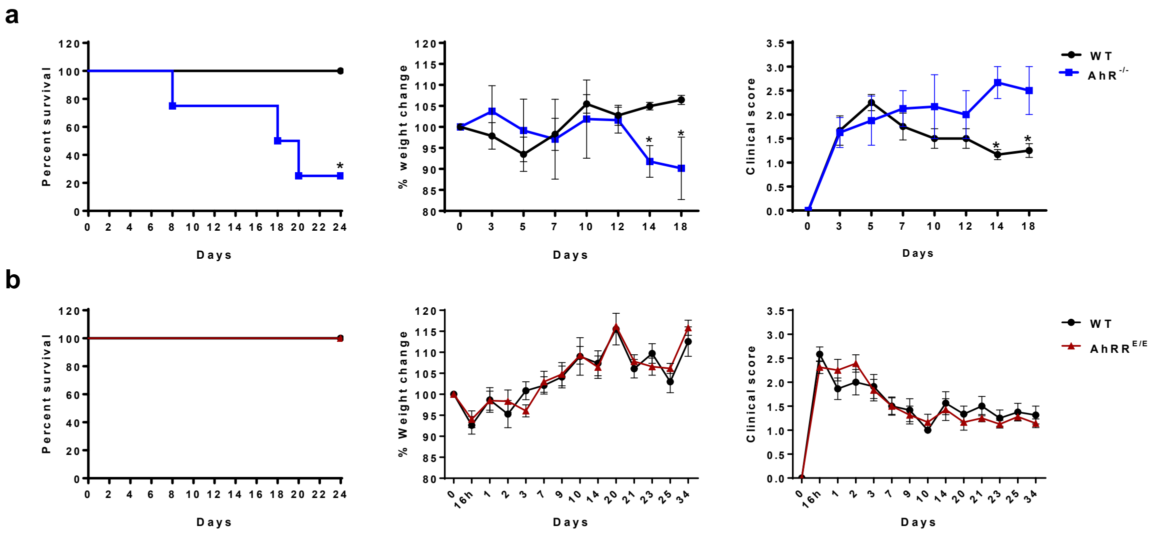

Supplementary Figure 7: AhRR-deficient mice are not susceptible to *C. rodentium* infection

(a) AhR-deficient and AhR wild type littermate controls (n= 4-6 mice of 2 independent experiments), **(b)** AhRR$^{E/E}$ mice and AhRR wildtypelitter mate controls (n= 11-13 mice of 3 independent experiments) were orally infected with 10^9-10^{10} CFU *C. rodentium*. Survival, weight change and clinical score were monitored. For determination of the clinical score, weight change, consistency of stool samples and macroscopic detection of rectal bleeding were analyzed and scored. Data are shown as mean ± s.e.m. and significance was determined by Log-rank (Mantle Cox) test and students *t*-test **p* <0.05, (WT controls vs AhR$^{-/-}$).

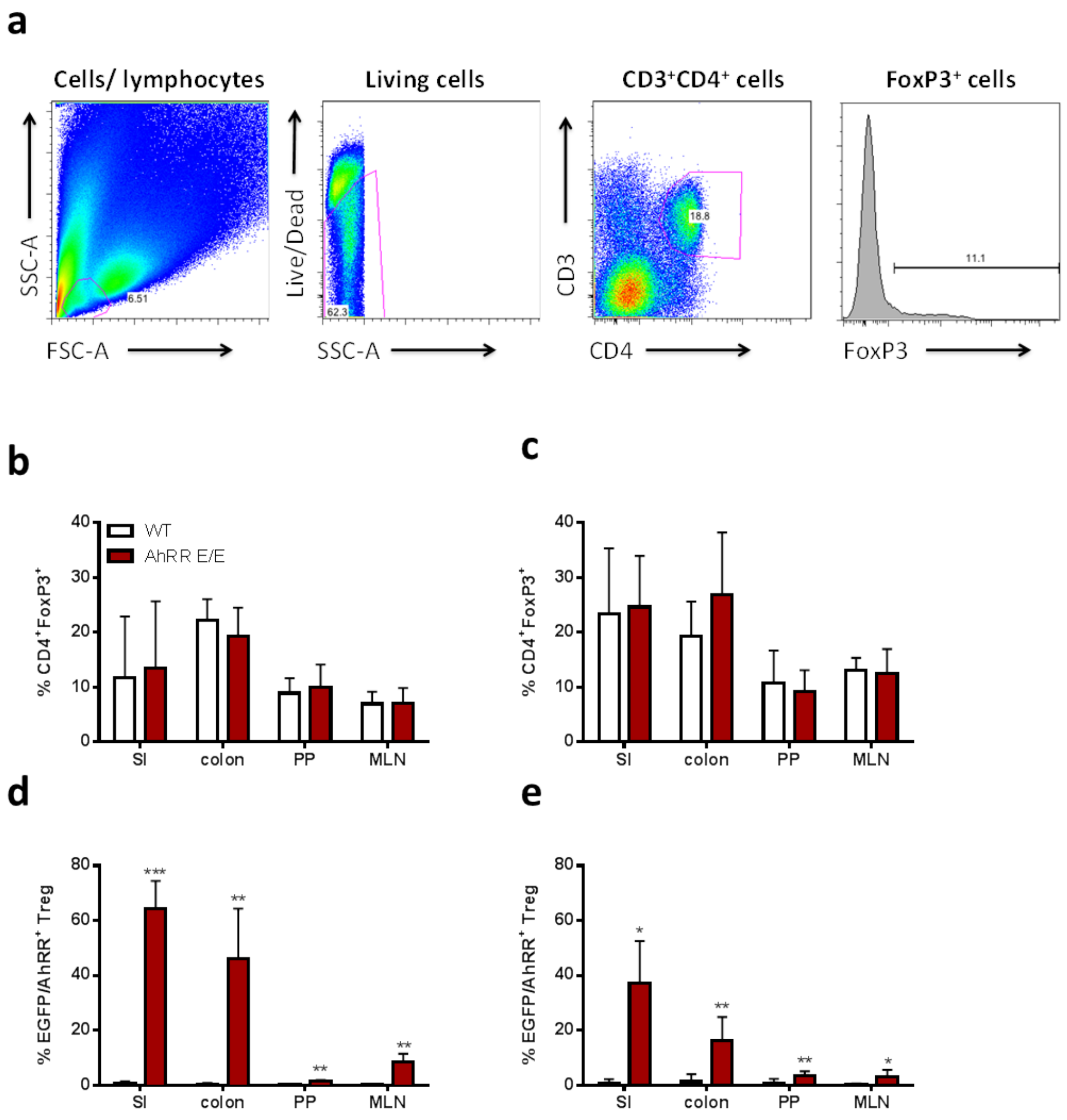

Supplementary Figure 8: Comparable frequencies of Treg in naïve and DSS-treated WT, AhRR$^{E/E}$ mice

(a) Gating strategy. Frequencies of Treg in the SI, colon, PP and MLN of **(b)** naive WT and AhRR$^{E/E}$ mice (n=6-7 mice of 3 independent experiments) or **(c)** DSS-treated WT, AhRR$^{E/E}$ mice (n=6 mice of 3 independent experiments). Frequencies of AhRR/EGFP expressing Treg in SI, colon, PP and MLN of **(d)** naïve and **(e)** DSS-treated WT, AhRR$^{E/E}$ and AhR$^{-/-}$ mice. Data are shown as mean ± s.d. and significance was determined by students *t*-test **p* <0.05, ***p* < 0.01, ***p< 0.001 (AhRR$^{E/+}$ controls vs AhRR$^{E/+}$ 3MC).

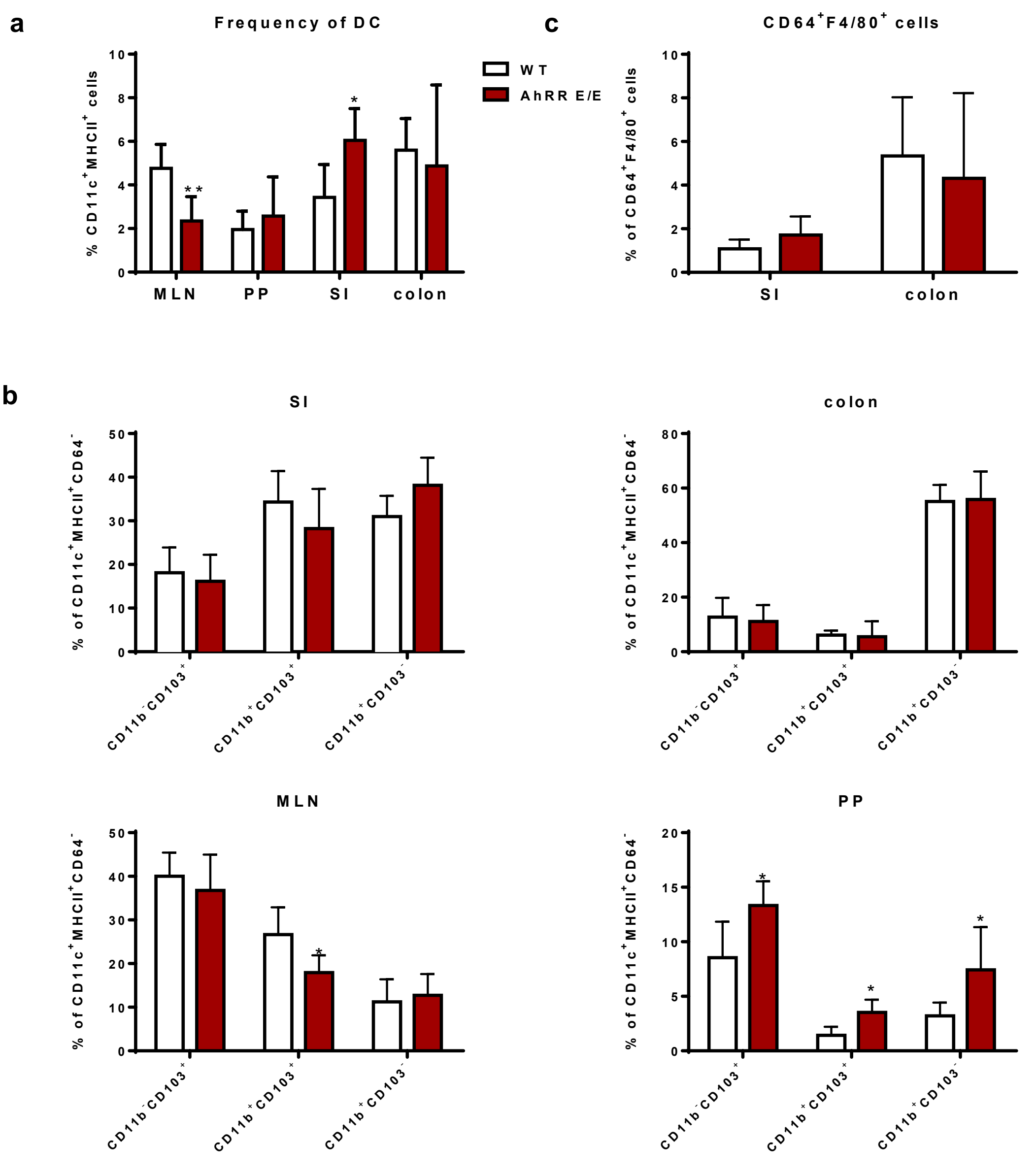

Supplementary Figure 9: Frequencies of myeloid cell populations in DSS-treated WT- and AhRR$^{E/E}$ mice

(a) Frequencies of CD11c^{+}MHCII^{+} cells in MLN, PP, SI and colon of DSS treated WT and AhRR$^{E/E}$ mice on day 6. **(b)** Frequencies of CD11b^{-}CD103^{+}, CD11b^{+}CD103^{+} and CD11b^{+}CD103^{-} DC subpopulations of CD11c^{+}MHCII^{+}cells in MLN, PP, SI and colon. **(c)** Frequencies of CD64^{+}F480^{+} macrophages in SI and colon of DSS treated mice on day 6 (n=5-

6 mice). Data are shown as mean ± s.d. and significance was determined by students *t*-test $**p < 0.01$, $***p < 0.001$ ($AhRR^{E/E}$ vs WT).
